# Comparative effects of angiotensin II stimulating and inhibiting antihypertensives on dementia risk: a systematic review and meta-analysis

**DOI:** 10.1007/s11357-025-01600-1

**Published:** 2025-04-04

**Authors:** Eyayaw Ashete Belachew, Gregory M. Peterson, Woldesellassie M. Bezabhe

**Affiliations:** 1 School of Pharmacy and Pharmacology, College of Health and Medicine, University of Tasmania, Private Bag 26, Hobart, TAS 7001 Australia; 2 Department of Clinical Pharmacy, School of Pharmacy, College of Medicine and Health Sciences, University of Gondar, Gondar, Ethiopia

**Keywords:** Antihypertensive medications, Angiotensin II stimulating, Dementia, Alzheimer’s disease, Vascular dementia, Mild cognitive impairment

## Abstract

Studies comparing the effects of Angiotensin II (Ang-II) stimulating and inhibiting antihypertensive medications (AHMs) on dementia risk have reported inconsistent findings. Based on the PRISMA guidelines, this study was performed to pool these findings. We searched PubMed, Scopus, Embase Ovid, PsycINFO, and CINAHL from inception to 22 May 2024 for randomised controlled trials (RCTs) and observational studies that compared the use of Ang-II stimulating (thiazides, Ang-II receptor blockers, and dihydropyridine calcium channel blockers) and inhibiting AHMs (β-blockers, angiotensin-converting enzyme inhibitors, and non-dihydropyridine calcium channel blockers) and the subsequent risk of developing dementia. Two reviewers independently performed study selection, data extraction, and quality assessment. Random effects meta-analysis models were used to calculate hazard ratios (HRs) or risk ratios (RRs) with their confidence intervals (CIs). All-cause dementia was the primary outcome. Alzheimer’s disease (AD), vascular dementia (VD), and mild cognitive impairment (MCI) were secondary outcomes. We included 18 studies with 1,883,283 participants. Observational studies showed that the use of Ang-II stimulating AHMs reduced the risk of all-cause dementia by 13% (HR = 0.87; 95% CI = 0.82–0.93) compared with Ang-II inhibiting AHMs. The risk of AD was reduced by 12% (HR = 0.88; 95% CI = 0.86–0.90), VD by 19% (HR = 0.81; 95% CI = 0.72–0.91), and MCI by 24% (HR = 0.76; 95% CI = 0.68–0.85) in these studies. A meta-analysis of four RCTs revealed a non-significant 8% reduction in dementia risk with Ang-II stimulating AHMs versus control (RR = 0.92; 95% CI = 0.79–1.08). Observational evidence suggests that Ang-II stimulating AHMs may offer neuroprotective benefits relative to Ang-II inhibiting AHMs.

**Supplementary Information:**

The online version contains supplementary material available at 10.1007/s11357-025-01600-1.

## Introduction

Midlife hypertension increases the risk of vascular dementia (VD) by approximately 60% [1] and Alzheimer’s disease (AD) by 25% [2]. Given the effectiveness and accessibility of current hypertension treatments [3], optimising the use of antihypertensive medications (AHMs) could be a potentially attractive strategy for reducing the risk of dementia.

Studies have found that AHMs are associated with a lower risk of developing dementia or mild cognitive impairment (MCI) [4, 5]. Their dementia preventive effect appears to be independent of their impact in lowering blood pressure (BP) [6, 7] and varies across classes of AHMs. The mechanisms are not fully understood. One commonly suggested mechanism involves the renin-angiotensin system (RAS), referred to as the angiotensin hypothesis [8–11]. Angiotensin II (Ang-II) lowers BP by activating angiotensin type 1 (AT1) receptors [12, 13], protects against ischaemia [12, 13] by activating AT2, and preserves memory by acting on AT4 [14, 15]. Angiotensin-converting enzyme increases degradation of amyloid-β (Aβ) protein; its accumulation drives the pathogenesis and progression of AD [16]. AHMs that increase and decrease Ang-II activity at AT2 and AT4 receptors are categorised as Ang-II stimulating and Ang-II inhibiting, respectively. The Ang-II stimulating group includes thiazide-type diuretics, Ang-II receptor blockers (ARBs), and dihydropyridine calcium channel blockers (DHP CCBs). Beta-blockers (BBs), angiotensin-converting enzyme inhibitors (ACEIs), and non-dihydropyridine calcium channel blockers (non-DHP CCBs) are classified as Ang-II inhibiting [17, 18].

Since the proposition of the angiotensin hypothesis, individual studies that compared the effects of Ang-II stimulating and inhibiting AHMs in reducing the risk of dementia or MCI have found inconsistent results [4, 9, 19–21]. Van Dalen et al. found a 43% lower incidence of dementia (hazard ratio (HR) = 0.57; 95% confidence interval (CI) = 0.34 to 0.89) among individuals using Ang-II stimulating AHMs compared to those using Ang-II inhibiting AHMs [18]. Marcum et al. reported that Ang-II stimulating AHMs reduced the risk of MCI by over a quarter (HR = 0.74; 95% CI = 0.64–0.87) compared to Ang-II inhibiting AHMs [22]. However, Schroeder et al. [23] found no significant difference in the risk of dementia between patients receiving Ang-II stimulating AHMs compared with patients receiving other AHMs (HR = 0.80; 95% CI = 0.61–1.04). Cohen et al. [20] reported no significant difference in risk of dementia or MCI in patients receiving Ang-II stimulating AHMs compared with patients taking Ang-II inhibiting AHMs (HR = 0.90; 95% CI = 0.72–1.12). These studies’ inconsistent findings and their differences in sample sizes and demographics make it difficult to draw definitive conclusions [20, 22–26]. A comprehensive review and quantitative synthesis of the evidence are therefore necessary.

To our knowledge, there has been no published systematic review of studies that compared the dementia risk reduction effects of Ang-II stimulating and inhibiting AHMs. We therefore conducted a systematic review and meta-analysis of published studies that compared the effects of Ang-II stimulating and inhibiting AHMs in reducing the risk of all-cause dementia or MCI.

## Methods

The study protocol was registered with PROSPERO (CRD42024542844) [27]. We carried out this systematic review and meta-analysis in accordance with the Preferred Reporting Items for Systematic Reviews and Meta-Analyses (PRISMA) guidelines [28, 29].

### Search strategy and data sources

We structured the search terms, keywords, and controlled vocabulary terms, which were focused on three concepts, using the PEO (patient, exposure, outcome) framework with the help of a research librarian. The first concept terms were related to BP and hypertension. The second concept terms were related to exposure to AHM (ARBs, ACEIs, diuretics, CCBs, and BBs). The third concept terms were related to dementia, AD, cognition, and cognitive impairment. We used the Boolean operator ‘OR’ to combine the terms within each concept and ‘AND’ to integrate the three concepts into the main search (Table S1). Five databases (PubMed, Scopus, Embase Ovid, PsycINFO, and CINAHL Complete) were searched from inception to 22 May 2024. We also carried out a citation analysis by looking through the reference lists of the included studies and by checking the first 100 hits with Google Scholar. Seven authors of studies with incomplete data were contacted. Of these, five responded, and one provided data that was included in the review [30].

### Eligibility

Based on the angiotensin hypothesis [17, 18, 22, 24], we grouped exposure to AHMs into four categories: Ang-II stimulating (exposure to at least one Ang-II stimulating AHM but no Ang-II inhibiting AHMs), Ang-II inhibiting (exposure to at least one Ang-II inhibiting AHM but no Ang-II stimulating AHMs), mixed AHMs (exposure to both Ang-II stimulating and Ang-II inhibiting AHMs), and other AHMs (exposure to AHMs that do not affect the RAS).

RCTs and observational studies focused on patients with hypertension were selected if they examined the association between the development of all-cause dementia, AD, VD, or MCI and the prior use of the four specified AHM groups, either in comparison with each other or with placebo/control groups. Studies were excluded if the mean or median age of the patients included was < 45 years or if they included patients with dementia or cognitive impairment at baseline. Studies were also excluded if they had a follow-up duration of less than 1 year and did not clearly define the relationship between AHM exposure and outcomes. Studies not published in English, editorials, commentaries, conference abstracts, opinions, research protocols, case reports, series of case reports, clinical guidelines, theses, letters to editors, qualitative studies, and case studies were also excluded.

### Screening and data extraction

All retrieved records were uploaded into Covidence software, where duplicate entries were removed. Two authors independently screened the titles, abstracts, and full texts. EAB screened all articles, while GP and WB screened half each. During the screening process, disagreements occurred in 254 out of 3635 articles (7%). These disagreements were primarily related to the interpretation of inclusion criteria and unclear reporting of study outcomes. All disagreements were resolved through discussion between the reviewers. Data extraction was carried out independently by two authors, EAB and WB, using a standardised data extraction form. Discrepancies during data extraction occurred in two out of 18 articles (11%), primarily due to unclear reporting of follow-up durations. These issues were resolved through discussion, ensuring consistency and accuracy in the final dataset. Extracted data encompassed study characteristics (first author, publication year, country, design, sample size, setting, aim), participant characteristics (age, gender, comorbidities), intervention details (criteria, AHM categories, follow-up duration), outcomes and outcome measures (diagnostic tools, incidence of dementia, AD, MCI, or MCI incidence), and model adjustment variables.

### Outcomes

The primary outcome was all-cause dementia. It was determined through medical records, regardless of the coding scheme used, or through diagnosis by licenced health professionals (doctors or neurologists) following standardised criteria [31–39]. The secondary outcomes were AD, VD, and MCI.

### Quality assessment

We employed the Joanna Briggs Institute (JBI) critical appraisal tools for evaluating the quality of RCTs [40] and cohort studies [41]. Two reviewers independently conducted the appraisals (EAB appraised all the studies, GP and WB appraised half each). Disputes between the two primary reviewers occurred in five out of 18 articles (28%), primarily due to differences in the interpretation of randomisation procedures, adequacy of follow-up, and the reliability of outcome measurement. No systematic differences in the direction of interpretation between the reviewers were observed. Disagreements generally stemmed from ambiguities in reporting study methods or borderline cases. A third reviewer was involved when disputes occurred, and consensus was achieved in all cases following discussion. We calculated the percentage of questions with favourable responses for each study included in our review. Based on these results, studies were classified as low (scores below 50%), moderate (scores ranging from 50 to 70%), or high quality (scores exceeding 70%) [42].

### Data synthesis and analysis

We used descriptive statistics to summarise the characteristics of the studies. Continuous data were reported using medians with interquartile ranges (IQR) or means with standard deviations (SD). We performed meta-analyses for both primary and secondary outcomes. The intention was to conduct separate meta-analyses for observational studies and RCTs to account for differences in study design with inherent variation in methodological rigour and potential biases, which may influence the pooled outcomes. Ultimately, irrespective of this, separate meta-analyses were required as studies with different designs did not have the same comparator groups. For the primary outcome, we conducted three analyses: [1] observational studies that compared Ang-II stimulating AHMs with Ang-II inhibiting ones, (2) RCTs comparing Ang-II stimulating AHMs with placebo/control, and (3) observational studies comparing mixed AHMs with Ang-II inhibiting AHMs. For the secondary outcome, we analysed AD, VD, and MCI separately. Since only one RCT study was available to compare Ang-II inhibiting AHMs with placebo [43] and only one observational study was available to compare Ang-II stimulating AHMs with other AHMs [23], these results were summarised descriptively.

We used HRs for observational studies and risk ratios (RRs) for RCTs to estimate pooled effects [44]. To mitigate confounding biases commonly associated with observational studies, such as treatment selection bias and insufficient reporting of concomitant medications, we selected the most adjusted HRs in the studies. For studies that did not report HRs or RRs, we calculated these measures using the sample size and number of events for each AHM class. A random effects model with inverse variance weighting was employed to compute the combined estimate for each outcome [45], HRs with 95% CIs for cohort studies and RRs with 95% CIs for RCTs. The logarithms of HRs and RRs, along with their standard errors, were derived from the HRs, RRs, and 95% CIs extracted from the studies. The heterogeneity across studies was quantified by using Higgins’ *I*^2^ statistic and Cochran’s *Q* [46]. Heterogeneity was deemed significant if the *I*^2^ value exceeded 60% [45]; considering that *I*^2^ can introduce bias in small meta-analyses [47], we adopted a comprehensive approach to identify and address heterogeneity. Subgroup and sensitivity analyses were conducted to explore variations by age, sex, comorbidities, and follow-up duration. These analyses aimed to identify sources of variation and assess the robustness of pooled estimates.

The sensitivity and subgroup analyses were performed for the meta-analyses of the primary outcome, all-cause dementia, comparing Ang-II stimulating and inhibiting AHM user groups. Sensitivity analyses were carried out with the leave-one-out method to assess the impact of each study on the pooled HRs. We used subgroup analyses to examine heterogeneity and how treatment effects varied across specific groups based on follow-up duration (≥ 6.8 vs < 6.8 years), adjustments for confounding (unadjusted vs adjusted HRs), baseline age (≥ 71 vs < 71 years), percentage of females (≥ 55% vs < 55%), AHM class exposure (ARBs vs ARBs, thiazides, and DHP CCBs vs DHP CCBs), country (USA vs Europe), and study design (retrospective cohort vs prospective cohort). Additionally, we also performed subgroup analyses by grouping studies based on the proportion of patients with comorbidities at baseline (diabetes mellitus (DM) (≥ 22% vs < 22%), stroke (≥ 7% vs < 7%), and coronary artery disease/ischaemic heart disease (CAD/IHD) (≥ 21% vs < 21%)). Data were insufficient to perform similar subgroup analyses based on congestive heart failure (CHF), systolic blood pressure (SBP) values, apolipoprotein E4 (APOE4) status, and statin co-medication use. The cutoff points were determined using the median value of each continuous variable. This approach ensured a balanced, clinically relevant data division, facilitating subgroup comparisons that reflected typical patterns while minimising bias from extreme values [48]. Publication bias in the meta-analyses comparing the risk of all-cause dementia between Ang-II stimulating and Ang-II inhibiting AHMs was assessed through visual inspection, Egger’s test [49], and the trim-and-fill method [50].

Pooled values were presented as point estimates with 95% CIs and evaluated for statistical significance with a threshold of *p* < 0.05. All the analyses were carried out using STATA (version 18.0, Stata Corp., College Station, TX, USA).

## Results

### Study selection

**Fig. 1:**
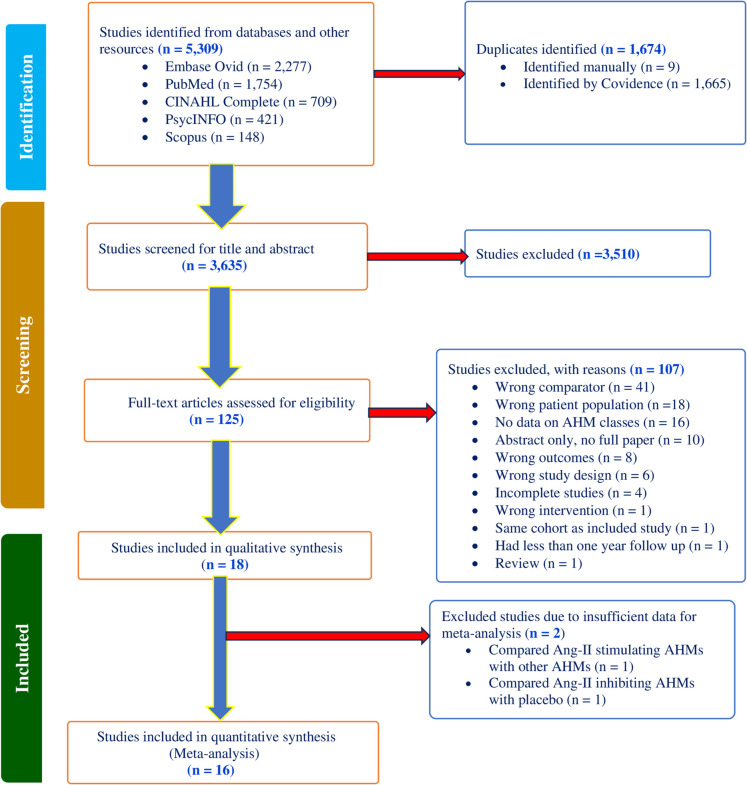
PRISMA flow chart of the study selection process and reasons for study exclusion (Ang-II, angiotensin II; AHM, antihypertensive medication)

The selection of the studies is shown in Fig. 1. We included 18 studies involving 1,883,283 patients.

### Study and participants’ characteristics

Of the 18 studies included, 13 were observational studies [18–20, 22–26, 51–55], and five were RCTs [30, 43, 56–58], with 596,182 participants in the exposure group (combined total across all four exposure groups) (range 78 [55] to 380,606 [19]) and 1,287,101 participants in the reference group (combined across all reference groups) (range 104 [55] to 640,715 [19]). The incidence of dementia was 11,864 out of 212,127 (5.6%) in the exposure group, compared to 22,538 out of 643,433 (3.5%) in the reference group. For MCI, the incidence was 446 out of 3371 (13.2%) in the exposure group and 545 out of 2849 (19.1%) in the reference group. Table 1 provides an overview of the studies.

**Table 1:** Characteristics of the studies included in the review

Author, year, and country	Study design and settings	Databases or trials	Age, mean (SD), years	Females, *n* (%)	AHM class (category) (E/R)^‡^	Sample size	Events, *n* (%)	Follow-up, median (IQR), years	Outcome and findings: RR/HR/, 95%CI
Barthold et al. 2018 [19], USA	Retrospective cohort, healthcare databases	Medicare claims data	78.3	671,279 (67)	ARBs (Ang^+^)	380,606 (1,034,502 P-Y)	8379 (8.1)^§^	7 (maximum)	AD, aHR (ref. Ang^−^) Ang^+^: 0.88 (0.86–0.90)*
					ACEIs (Ang^−^)	640,715 (1,764,546 P-Y)	16,233 (9.2)^§^		
Cohen et al. 2022 [20], USA	Prospective cohort, clinical settings	SPRINT trial	67.2 (9.5)	749 (38)	ARBs (Ang^+^)	727	103 (3.8)	4.9	Mild cognitive impairment, aHR (ref. Ang^−^) Ang^+^: 0.90 (0.72–1.12)
					ACEIs (Ang^−^)	1313	233 (4.3)		
Colbourne et al. 2022 [51], UK	Prospective cohort, healthcare databases	TriNetX electronic health records network	59.8 (17.0)	56,992 (57)	DHP CCBs^a^ (Ang^+^)	49,987	549 (1.1)	2	All-cause dementia, RR (ref. Ang^−^) Ang^+^: 1.03 (0.92–1.16)
					Non-DHP CCBs^b^ (Ang^−^)	49,987	499 (1)		
Diener et al. 2008 [56], across continents	RCT, clinical settings	PRoFESS trial	66.1 (8.6)	7310 (36)	Telmisartan (ARB) (Ang^+^)	8624	408 (4.7)	2.4	All-cause dementia, RR (ref. Placebo) Ang^+^: 1 (0.87–1.15)*
					Placebo	8646	409 (4.7)		
Du et al. 2023[24], USA	Retrospective cohort, healthcare databases	Medicare linked database	Median 76.3 (IQR, 65–108)	32,205 (54.8)	Thiazides, ARBs, DHP CCBs (Ang^+^)	7937	1722 (21.7)	10 (maximum)	All-cause dementia, aHR (ref. Ang^−^) Ang^+^: 0.82 (0.78–0.87) Ang^±^: 1.18 (1.13–1.24)*
					Non-DHP CCBs, ACEIs, BBs (Ang^−^)^**‡**^	14,052	3274 (23.5)		
					Mixed (Ang^±^)	11,504	3313 (28.2)		
Goh et al. 2014 [52], UK	Prospective cohort, healthcare databases	CPRD	Median 64.5 (IQR, 54–74)	225,295 (48)	ARBs (Ang^+^)	45,541	664 (1.5)	4.25 (2.51–6.65)	All-cause dementia, aHR (ref. Ang^−^) Ang^+^: 0.92 (0.85–1.00)
					ACEIs (Ang^−^)	380,548	5853 (1.5)		
Hu et al. 2020 [30], China	RCT, clinical settings	ClinicalTrials.gov and ChiCTR.org.cn	70.30 (6.19)	298 (47.9)	Telmisartan (ARBs) (Ang^+^)	311	42 (13.5)^$^	7 (6.7–7.2)	All-cause dementia, RR (ref. Placebo) Ang^+^: 0.63 (0.44–0.90)
					Placebo	311	72 (23.1)^$^		
Hwang et al. 2016 [53], Korea	Prospective cohort, insurance database	KNHIS	67.22	769 (56.2)	DHP CCBs (Ang^+^)	11,816	2444 (20.7)	Average 9.4	All-cause dementia, HR (ref. Ang^−^) Ang^+^: 0.92 (0.70–1.20)*
					Non-DHP CCBs (Ang^−^)	269	54 (20.1)		
Li et al. 2010 [54], USA	Prospective cohort, healthcare databases	Veterans’ health system database	74 (5.5)	14,383 (1.8)	ARBs (Ang^+^)	11,507	395 (3.4)	Average 2.5	All-cause dementia, aHR (ref. Ang^−^) Ang^+^: 0.81 (0.73–0.90)
					ACEIs (Ang^−^)	91,164	3768 (4.1)		
Lithell et al. 2004 [57], USA	RCT, clinical settings	SCOPE trial	76.4 (4.5)	1390 (66.3)	Candesartan (ARBs) (Ang^+^)	1253	31 (2.5)	Ang^+^: Mean 3.7 Placebo: Mean 3.5	All-cause dementia, RR (ref. Placebo) Ang^+^: 1.16 (0.65–2.09)*
					Placebo	845	18 (2.1)		
Marcum et al. 2022 [22], USA	Prospective cohort, clinical settings	SPRINT trial	67.9 (9.3)	1484 (34.35)	ARBs, DHP CCBs, Thiazides (Ang^+^)	2644	373 (14.1)	4.8 (4.7–4.8)	Mild cognitive impairment, aHR (ref. Ang^−^) Ang^+^: 0.74 (0.64–0.87)
					ACEIs, BBs, Non-DHP CCBs (Ang^−^)	1536	312 (20.3)		
Marcum et al. 2023 [25], USA	Retrospective cohort, healthcare databases	Medicare beneficiaries	73.8 (6.3)	36,348 (62.9)	Thiazides, ARBs, DHP CCBs (Ang^+^)	4879	1425 (29.2)	6.9 (4.7–9.3)	All-cause dementia, aHR (ref. Ang^−^) Ang^+^: 0.84 (0.75–0.90) Ang^±^: 0.90 (0.84–0.96)
					Non-DHP CCBs, ACEIs, BBs (Ang^−^)^**‡**^	10,303	3266 (31.6)		
					Mixed (Ang^±^)	2179	1244 [57]		
Schroeder et al. 2023 [23], The Netherlands	Prospective cohort, clinical settings	PreDIVA trial	74.5 (2.5)	1027 (53.9)	Thiazides, ARBs, DHP CCBs (Ang^+^)	1180	129 (10.9)	10.4 (6.8–11)	All-cause dementia, aHR (ref. Ang^−^) Ang^+^: 0.80 (0.61–1.04)
					Other AHMs	727	96 (13.2)		
Schroeder et al. 2024 [26], The Netherlands	Retrospective cohort, healthcare databases	General practice registration networks	Median 68.2 (IQR, 62.0–75.8)	72,884 (54.7)	Thiazides, ARBs, DHP CCBs (Ang^+^)	63,026(631,650 P-Y)	3491 (5.5)^§^	7.6 (4.1–11.0)	All-cause dementia, aHR (ref. Ang^−^) Ang^+^: 0.88 (0.82–0.95)
					Non-DHP CCBs, ACEIs, BBs (Ang^−^)	80,305(727,829 P-Y)	4312 (5.9)^§^		
Tzourio et al. 2003 [43], across continents	RCT, clinical settings	PROGRESS trial	64 (10)	768 (30)	Perindopril (ACEIs) (Ang^−^)	1281	87 (6.7)	Mean 3.9	All-cause dementia, RR (ref. Placebo) Ang^−^: 1.06 (0.79–1.43)
					Placebo	1280	81 (6.3)		
Van Dalen et al. 2021[18], The Netherlands	Prospective cohort, clinical settings	PreDIVA trial	74.5 (2.5)	1025 (53)	ARBs, DHP CCBs, Thiazides (Ang^+^)	480	27 (5.6)	6.7 (5.8–7.0)	All-cause dementia, aHR (ref. Ang^−^) Ang^+^: 0.57 (0.35–0.93) Ang^±^: 0.77 (0.51–1.15)
					ACEIs, BBs, Non-DHP CCBs (Ang^−^)^**‡**^	721	59 (8.2)		
					Mixed (Ang^±^)	669	46 (6.8)		
Whiteley et al. 2021 [58], UK	RCT, clinical settings	ASCOT trial	64 (8)	1620 (18.9)	Amlodipine based (DHP CCBs) (Ang^+^)	4305	450 (10.45)	5.5	All-cause dementia, aRR (ref. Control) Ang^+^: 0.94 (0.82–1.05)
					Atenolol based (BBs) (Control)	4275	465 (10.9)		
Yasar et al. 2005 [55], USA	Prospective cohort, research centre	Baltimore longitudinal study of ageing	80.5	407 (37.3)	DHP CCBs (Ang^+^)	78	6 (7.7)	Mean 11.0 (range 0.3–19.5)	AD, HR (ref. Ang^−^) Ang^+^: 0.69 (0.27–1.77)*
					Non-DHP CCBs (Ang^−^)	104	12 (11.5)		

Abbreviations: *95% CI*, 95% confidence interval; *Ang*^*+*^, angiotensin II stimulating medication; *Ang*^*−*^, angiotensin II inhibiting medication; *Ang*^*±*^, both angiotensin II stimulating and angiotensin II inhibiting medication; *ACEIs*, angiotensin-converting enzyme inhibitors; *AD*, Alzheimer’s disease; *ADRD*, Alzheimer’s disease and related dementias; *AHMs*, antihypertensive medications; *Ang-II*, angiotensin II; *ARBs*, angiotensin II receptor blockers; *ASCOT*, Anglo-Scandinavian Cardiovascular Outcomes Trial; *BBs*, beta-blockers; *ChiCTR.org.cn*, Chinese Clinical Trial Registry; *DHP CCBs*, dihydropyridine calcium channel blockers;*E/R*, exposure/reference; *HR*, unadjusted hazard ratio; *aHR*, adjusted hazard ratio; *IQR*, interquartile range;*KNHIS*, Korean National Health Insurance Service Database; *SPRINT*, Systolic Blood Pressure Intervention Trial; *P-Y*, person-years; *PRoFESS*, Prevention Regimen For Effectively avoiding Second Strokes; *PROGRESS*, Perindopril Protection Against Recurrent Stroke Study; *RCT*, randomised controlled trial; *ref*, reference; *RR*, unadjusted risk ratio; *aRR*, adjusted risk ratio; *USA*, United States of America

Other AHMs; use of antihypertensive medications that do not affect the RAS, ^‡^Reference group: the last row is used as the reference group unless otherwise specified; ^§^Incidence rate per 1000 person-years at risk; ^$^This data was obtained from the corresponding authors of the study; *RRs and HR are calculated by the authors; ^a^97% of the participants used nifedipine, felodipine, and nicardipine; ^b^35% on verapamil, 67% on diltiazem

Most of the included studies were from the USA (*n* = 8) [19, 20, 22, 24, 25, 54, 55, 57] and Europe (*n* = 6) [18, 23, 26, 51, 52, 58]. The remaining studies were conducted across continents (*n* = 2) [43, 56], with two studies in Asia (China [30] and Korea [53]). Participants’ data were obtained from various sources: clinical settings (*n* = 9) [18, 20, 22, 23, 30, 43, 56–58], healthcare databases (*n* = 7) [19, 24–26, 51, 52, 54], research centres (*n* = 1) [55], and insurance databases (*n* = 1) [53]. The mean age of participants at baseline varied from 58.8 [51] to 80.5 years [55], and the percentage of female participants ranged from 1.8 [54] to 67% [19]. Baseline comorbidities and medication use history reported by the studies are detailed in Table S2.

Thirteen studies reported all-cause dementia [18, 23–26, 43, 51–54, 56–58], three focused solely on AD [19, 30, 55], and two examined only MCI [20, 22]. Of the 16 studies included in the meta-analysis, nine reported adjusted HRs [18, 20, 22, 24–26, 52, 54, 58], and one reported unadjusted HR [51]. For the remaining six studies [19, 30, 53, 55–57], unadjusted HRs and RRs were calculated from available data, i.e. HRs were computed from the number of events and person-years [19] and the number of events and total exposure [53, 55]. RRs were calculated based on the number of events and participants exposed to each treatment [30, 56, 57] (Table 1).

The median follow-up period varied from 2 [51] to 11 [58] years. Twelve observational studies compared Ang-II stimulating with Ang-II inhibiting AHMs [18–20, 22, 24–26, 51–55]. One observational study compared Ang-II stimulating AHMs with other AHMs [23], and four RCTs compared Ang-II stimulating AHMs with a placebo [30, 56–58]. One RCT compared Ang-II inhibiting AHMs with a placebo [43]. In terms of AHM classes of exposure, seven studies focused on ARBs [19, 20, 30, 52, 54, 56, 57], six studies on ARBs, thiazides, and DHP CCBs [18, 22–26], four studies on DHH CCBs [51, 53, 55, 58], and one study on ACEIs [43] (Table 1).

Four observational studies defined exposure to AHMs based on MPR ≥ 80% [24, 25, 53, 54]. One observational study defined exposure as having a 90-day supply along with a minimum of two drug claims annually for two consecutive years [19]. Another observational study required ≥ 3 successive prescriptions of an AHM class [26]. Two observational studies defined exposure based on baseline AHM use [18, 23], while five RCTs focused on consistent use of the same AHMs throughout the follow-up period [30, 43, 52, 56, 57]. Two observational studies defined AHM exposure as any use of AHMs during the follow-up period [52, 55]. The duration of AHM use was defined as at least 1 year in one observational study [20], at least 2 years in another observational study [51], and at least 6 months in a third observational study [22] (Table S3).

Dementia or MCI was identified using clinical diagnostic criteria (*n* = 10) [18, 20, 22, 23, 30, 43, 53, 55–57] or recorded coding (*n* = 8) [19, 25, 26, 51, 24, 52, 54, 58]. Specifically, three studies used the Diagnostic and Statistical Manual of Mental Disorders (DSM; fourth edition) [18, 23, 43]; two used the Montreal Cognitive Assessment [20, 22]; and seven relied on the International Classification of Diseases (9th or 10th revision) [19, 20, 24, 25, 54, 57, 58]. The remaining studies utilised the DSM (third edition, revised), the National Institute of Neurological and Communicative Disorders and Stroke-Alzheimer’s Disease and Related Disorders Association criteria [55], the International Classification of Primary Care [26], the Mini-Mental State Examination [30, 56], or specific read codes from the UK Clinical Practice Research Datalink [52] (Table S3).

### Quality assessment

Evaluation with the JBI tool revealed that the studies were of high quality, with each study scoring ≥ 70%, except for one study by Whiteley et al. [58], which was judged as moderate quality (Tables S4 and S5).

### Meta-analyses

Sixteen studies, 12 observational studies [18–20, 22, 24–26, 51–55] and four RCTs [30, 56–58], were included in the meta-analyses. Two studies (one RCT [43] and one observational study [23]) were excluded from the meta-analysis due to insufficient data.

#### All-cause dementia: Ang-II stimulating vs Ang-II inhibiting AHMs

**Fig. 2:**
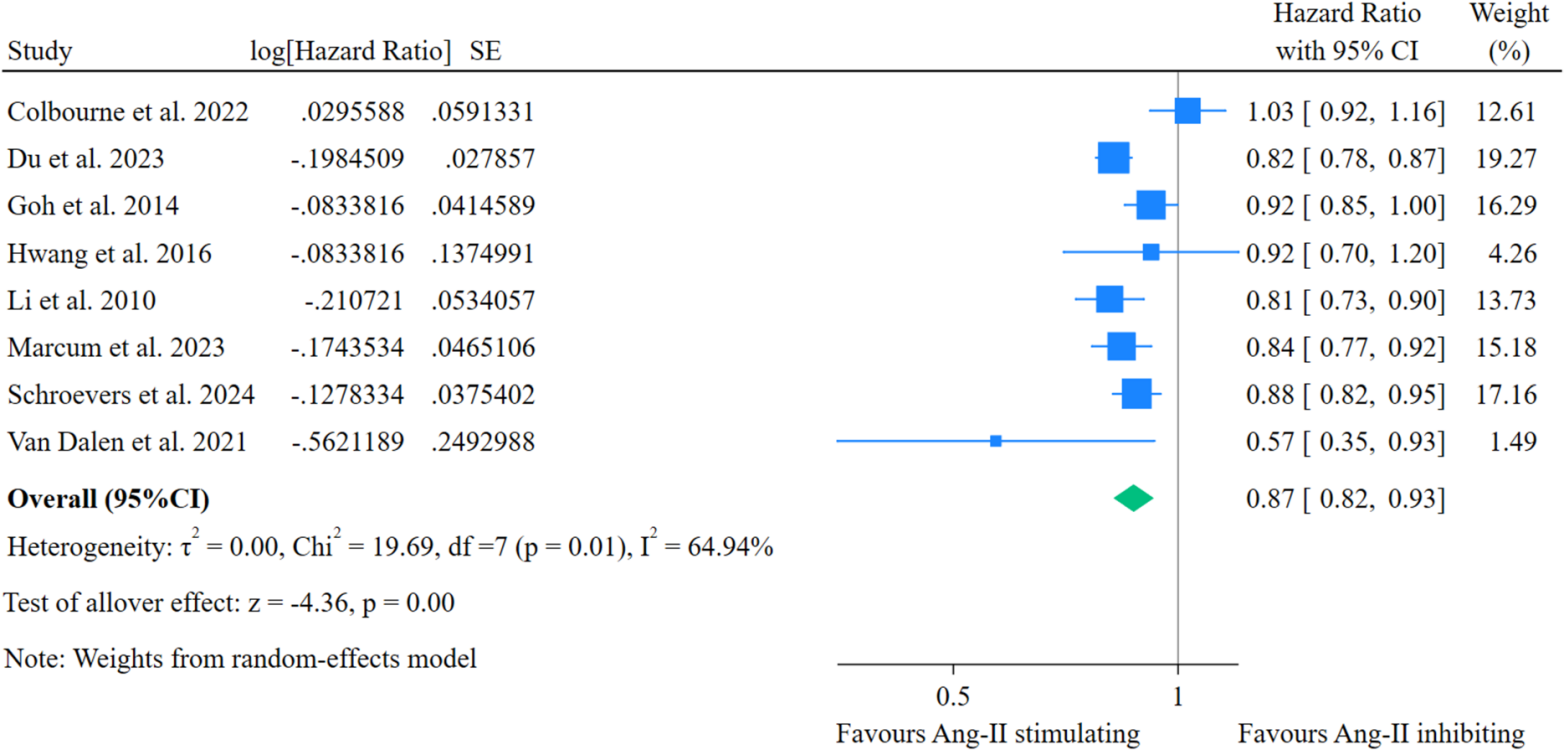
The incidence of all-cause dementia between Ang-II stimulating and inhibiting antihypertensive medication users

Eight observational studies [18, 24–26, 51–54] comparing Ang-II stimulating and Ang-II inhibiting AHMs were included. The results showed that the use of Ang-II stimulating AHMs was associated with a 13% decrease in the risk of all-cause dementia compared to Ang-II inhibiting AHMs (HR = 0.87; 95% CI = 0.82–0.93, *p* < 0.01) (Fig. 2).

##### Sensitivity analyses

High heterogeneity was observed among the studies (chi^2^ = 19.69, *p* = 0.01, *I*^2^ = 64.9%). The leave-one-out sensitivity analyses indicated that the study by Colbourne et al. [51] was a major contributor to this heterogeneity, and its exclusion reduced the *I*^2^ from 64.9 to 40.2% (Table S6). Excluding the study by Du et al. [24], which focused on colorectal cancer survivors with hypertension, reduced the *I*^2^ to 59.8% (Table S6). The dementia-preventive effect of Ang-II stimulating AHMs remained significant compared to Ang-II inhibiting AHMs in the leave-one-out analyses (Fig. S1).

##### Subgroup analyses

Subgroup analyses showed that Ang-II stimulating AHMs were associated with a significantly reduced risk of all-cause dementia compared with Ang-II inhibiting AHMs in studies that adjusted for confounders (HR = 0.85, 95% CI = 0.81–0.89, *p* < 0.001, *n* = 6) [18, 24–26, 52, 54], but not in those studies that did not adjusted for confounders (HR = 1.01, 95% CI = 0.91–1.13, *p* = 0.83, *n* = 2) [51, 53] (Fig. S2). In the subgroup meta-analysis of four studies with a follow-up duration ≥ 6.8 years, we found that Ang-II stimulating AMHs were associated with a significantly reduced risk of all-cause dementia (HR = 0.84, 95% CI = 0.81–0.88, *p* < 0.001) [24–26, 53] compared with Ang-II inhibiting AHMs. However, this was not significant in the meta-analysis of four studies with follow-ups < 6.8 years (HR = 0.88, 95% CI = 0.76–1.02, *p* = 0.10) [18, 51, 52, 54] (Fig. S3). Significant effects were observed for those exposed to ARBs (HR = 0.87, 95% CI = 0.77–0.98, *p* = 0.02, *n* = 2) [52, 54] and a combination of ARBs, thiazides, and DHP CCBs (HR = 0.84, 95% CI = 0.80–0.88, *p* < 0.001, *n* = 4) [18, 22, 24–26], but not for those exposed only to DHP CCBs (HR = 1.01, 95% CI = 0.91–1.13, *p* = 0.90, *n* = 2) compared to Ang-II inhibiting AHMs [51, 53] (Fig. S4).

Additional subgroup analyses were conducted by stratifying studies based on age (≥ 71 vs < 71 years, *n* = 8) [18, 24–26, 51–54], sex (percentage of women) (≥ 55% vs < 55%, *n* = 8) [18, 24–26, 51–54], observational study design (retrospective cohort vs prospective cohort, *n* = 8) [18, 24–26, 51–54], country (USA vs Europe, *n* = 7) [18, 24–26, 51, 52, 54], and baseline prevalence of comorbidities (DM (≥ 22% vs < 22%, *n* = 6) [19, 25, 26, 52–54], stroke (≥ 7% vs < 7%, *n* = 5) [18, 25, 26, 52, 54], and CAD/IHD (≥ 21% vs < 21%, *n* = 5) [18, 25, 26, 53, 54]). The effect sizes remained consistent across these subgroups (Table S7). Data were insufficient for subgroup analyses based on baseline APOE4 (*n* = 1) [18], statin co-medication (*n* = 3) [18, 25, 52], baseline SBP (*n* = 4) [18, 51, 53, 54], and CHF (*n* = 3) [25, 26, 52].

Visual inspection of the funnel plot (Fig. S6) revealed minimal asymmetry. Egger’s test (*p* = 0.44) and the trim-and-fill method (*p* = 0.143) also did not suggest publication bias.

#### All-cause dementia: Ang-II stimulating AHMs vs placebo/control

Four RCTs [30, 56–58] compared the dementia risk reduction effect of Ang-II stimulating AHMs with placebo or control. A meta-analysis of these studies showed an 8% reduction in the incidence of all-cause dementia with Ang-II stimulating AHMs. However, the reduction was statistically insignificant (RR = 0.92, 95% CI = 0.79–1.08, *p* = 0.30). Minimal heterogeneity was observed (chi^2^ = 6.05, *p* = 0.11, *I*^2^ = 50.4%) (Fig. S7).

#### All-cause dementia: mixed AHMs vs Ang-II inhibiting AHMs

Three observational studies [18, 24, 25] compared mixed AHMs with Ang-II inhibiting AHMs. The pooled estimate showed no significant difference in the incidence of all-cause dementia between users of mixed AHMs and Ang-II inhibiting AHMs (HR = 0.98, 95% CI = 0.77–1.24, *p* = 0.85), with significant heterogeneity (chi^2^ = 45.25, *p* < 0.01, *I*^2^ = 95.3%) (Fig. S8).

#### Alzheimer’s disease

Five observational studies [19, 24, 53–55] compared the effects of Ang-II stimulating and Ang-II inhibiting AHMs on AD risk reduction. The pooled estimate showed that Ang-II stimulating AHMs reduced the risk of AD by 12% compared to Ang-II inhibiting AHMs (HR = 0.88, 95% CI = 0.86–0.90, *p* < 0.01) (Fig. S9). This effect was influenced by Barthold et al. [19], which had a large sample size and dominated the results. In a leave-one-out analysis, when the Barthold et al. study was excluded from pooled estimation, the AD risk reduction increased to 15% (HR = 0.85, 95% CI = 0.77–0.94, *p* < 0.01) (Fig. S5).

#### Vascular dementia

Three observational studies [24, 25, 53] compared the association of Ang-II stimulating and Ang-II inhibiting AHMs with the risk of VD. The pooled estimate showed that using Ang-II stimulating AHMs reduced VD risk by 19% (HR = 0.81, 95% CI = 0.72–0.91, *p* < 0.01). No heterogeneity was observed among the included studies (chi^2^ = 0.65, *p* = 0.72, *I*^2^ = 0.00%) (Fig. S10).

#### Mild cognitive impairment

Three observational studies [20, 22, 24] compared the incidence of MCI between Ang-II stimulating and Ang-II inhibiting AHMs. The pooled estimate indicated that Ang-II stimulating AHMs were associated with a 24% reduced risk of MCI (HR = 0.76, 95% CI = 0.68–0.85, *p* < 0.01). There was no significant heterogeneity among the studies (chi^2^ = 0.28, *p* = 0.87, *I*^2^ = 0.00%) (Fig. S11).

#### Studies not included in the meta-analyses

The Schroevers et al. [23] observational study, which was not included in the meta-analysis, compared Ang-II AHMs with other AHMs. A small study found that Ang-II stimulating AHMs significantly reduced dementia risk over a median follow-up of 6.7 years (HR = 0.60; 95% CI = 0.37–0.98) compared to other AHMs. However, this protective effect diminished after 10.4 years (HR = 0.80; 95% CI = 0.61–1.04).

The RCT by Tzourio et al. [43], which was also not included in the meta-analysis, compared perindopril (an Ang-II inhibiting AHM) with a placebo. They found a slightly higher, though not statistically significant, dementia risk in the treatment group (6.7%) compared to the placebo group (6.3%) (RR = 1.06; 95% CI = 0.79–1.43, *p* = 0.69).

## Discussion

Hypertension is recognised as a potentially modifiable risk factor for dementia, and the use of AHMs probably reduces the incidence of dementia [1, 59]. However, the question of which AHMs are most effective in reducing this risk remains unresolved. Previous reviews have not categorised AHMs based on the angiotensin hypothesis, and their conclusions remain inconclusive [2, 5, 60, 61]. Since the introduction of the angiotensin hypothesis, individual studies that compared Ang-II stimulating and inhibiting AHMs have produced mixed and inconclusive results [20, 22, 23, 25]. To our knowledge, this is the first systematic review and meta-analysis of these individual studies. By doing so, our study fills an important gap in the literature, focusing specifically on the angiotensin hypothesis to evaluate the differential effects of various AHMs. This approach addresses inconsistencies found in previous studies [20, 22, 23, 25] and offers a comprehensive evaluation of Ang-II stimulating AHMs in reducing the risk of dementia or MCI, representing a significant and novel contribution to the field.

Our meta-analysis suggests that Ang-II stimulating AHMs may reduce the risk of all-cause dementia by 13% compared to Ang-II inhibiting AHMs. The potential dementia reduction effects of Ang-II stimulating AHMs may be linked to Ang-II’s role in dementia pathogenesis, supported by animal [62, 63] and human studies [64–66]. The differential effects of Ang-II stimulating and inhibiting AHMs could be due to their distinct impacts on Ang-II signalling. Ang-II inhibiting AHMs, particularly ACEIs, block both AT1 and AT2 receptors, raising bradykinin levels, which may contribute to vascular inflammation, oxidative stress, and neurodegeneration. Other Ang-II inhibiting AHMs, such as non-DHP CCBs and BBs, reduce renin release, which leads to a decrease in circulating Ang-II levels [11, 67, 68]. In contrast, Ang-II stimulating AHMs, particularly ARBs, selectively block AT1 receptors while simultaneously elevating Ang-II levels at AT2 and AT4 receptors via enhanced renin release. DHP CCBs and thiazide diuretics raise Ang-II levels at AT2 and AT4 without blocking AT1 receptors [11, 68, 69]. AT2 receptors have critical brain functions, with AT2-deficient mice showing impaired spatial memory and altered dendritic morphology [63]. In animal models, agonists of AT2 receptors improve memory and reduce brain damage, indicating their potential benefits in cognition [70] and mitigating cognitive deficits through increased neurogenesis, particularly in traumatic brain injury [71]. Additionally, Ang-II stimulating AHMs may enhance blood flow, lower the risk of microbleeding episodes, prevent lacunar infarcts, and reduce the development of white matter lesions through the activation of AT2 and AT4 receptors, as well as nicotinic acetylcholine receptors [14, 15, 72, 73].

The observed differences may also involve amyloid metabolism. Ang-II inhibiting AHMs, like ACEIs, increase Aβ levels by inhibiting its degradation [74, 75], potentially raising the risk of dementia or AD. In contrast, Ang-II stimulating agents may reduce amyloid pathology by activating the peroxisome proliferator-activated receptor gamma (PPAR-γ) pathway [76], which promotes Aβ degradation [77]. This could explain the lower amyloid burden and a slower decline in cerebrospinal fluid Aβ 1–42 levels in patients treated with Ang-II stimulating compared to other antihypertensives [65, 66].

Most subgroup and sensitivity analyses consistently showed a lower risk of dementia with Ang-II stimulating AHMs compared to Ang-II inhibiting AHMs. Longer follow-up duration revealed stronger dementia risk reduction effects of Ang-II stimulating AHMs compared to Ang-II inhibiting AHMs, likely due to the cumulative impact of AHMs on dementia risk [78]. Subgroup analyses based on AHM classes (ARBs vs ARBs, DHP CCBs, and thiazides vs DHP CCBs) suggested that the differences between Ang-II stimulating and inhibiting AHMs were mainly driven by thiazides and ARBs rather than DHP CCBs. While all classes were associated with a reduced dementia risk overall, DHP CCBs showed a slight, non-significant increase in risk in the subgroup analysis. The differing mechanisms of Ang-II stimulating AHMs may contribute to the observed effects. ARBs have potentially strong neuroprotective effects via AT1 receptors and renin activation, compared with the weaker renin influence and lack of AT1 receptor impact of DHP CCBs [79].

The four meta-analyses for the RCTs did not show significant differences in dementia risk between Ang-II stimulating versus placebo/control. However, when comparing these RCTs with observational studies, a discrepancy arises: observational studies suggested that Ang-II inhibiting AHMs may increase the risk of dementia. This discrepancy could be due to differences in their effects on the RAS. ACE inhibitors block angiotensin I to angiotensin II conversion and raise bradykinin levels, which leads to vascular inflammation, oxidative stress, and neurodegeneration [67]. They may also increase Aβ accumulation by inhibiting its degradation, potentially raising dementia risk [75]. The potential neuroprotective effects of Ang-II stimulating and inhibiting AHMs require further exploration, especially in light of the discrepancies between observational and RCT data. Only with additional high-quality and prolonged RCTs that account for potential confounders, including BP, and employing detailed mechanistic data, can the role of these medications in dementia risk be more definitively understood.

The strength of this systematic review and meta-analysis lies in its comprehensive analyses of data from 1,883,283 patients. Including population-based observational studies enhances the generalisability to real-world patients with hypertension. All observational studies included only subjects treated with AHMs, which helped limit potential confounding by including untreated subjects who might be healthier and at lower risk of dementia. The association between exposure to Ang-II stimulating AHMs, compared to Ang-II inhibiting AHMs, and a reduced risk of all-cause dementia remained consistent across most subgroup and sensitivity analyses. Additionally, visual inspection of the funnel plot revealed minimal asymmetry, with no significant publication bias detected.

Several limitations should be considered in this review. First, our primary findings comparing the risk of all-cause dementia between Ang-II stimulating and inhibiting groups were drawn from observational studies, which are prone to confounding by indication. To minimise bias, we used adjusted data from six studies [18, 24–26, 52, 54] in our primary analysis. Second, heterogeneity among the included studies was likely driven by differences in inclusion criteria, outcome identification, follow-up durations, and exposure definitions. A significant source of this heterogeneity was the study by Colbourne et al. [51], which focused on neuropsychiatric syndromes in a younger population and faced uncertainties regarding treatment adherence and continuous exposure over 2 years. However, leave-one-out analyses suggested that the effect sizes were consistent overall, implying that the Colbourne study did not disproportionately influence the overall findings. It is acknowledged that residual heterogeneity may remain. The results should, therefore, be interpreted with caution. Third, limited data availability constrained our ability to perform subgroup analyses based on factors such as baseline APOE4 status, CHF prevalence, SBP, and statin co-medication use. Fourth, the meta-analysis of RCTs should be interpreted with caution due to the inclusion of only four studies, each with varying placebo conditions and lacking detailed information on additional AHM use. Furthermore, these RCTs were not designed to investigate dementia as their primary objective. Fifth, the included papers lacked adequate BP control data, and we could not assess the impact of BP on dementia risk. Although BP-lowering effects are generally similar across AHM classes [80] and a meta-analysis found that BP reduction did not influence the association between AHM use and dementia risk [5, 80], the lack of BP data is a limitation. Lastly, some studies did not account for co-medication exposure when comparing Ang-II stimulating with Ang-II inhibiting AHMs [19, 51–55]. However, sensitivity analyses showed that the effect remained unchanged irrespective of whether studies clearly reported co-medication exposure.

## Conclusion and perspectives

This meta-analysis suggests that Ang-II stimulating AHMs, compared with Ang-II inhibiting AHMs, might lower the risk of dementia in hypertensive patients. However, the methodological limitations of this review, along with substantial heterogeneity across the included studies, warrant cautious interpretation of the findings. These findings could inform hypertension management guidelines and suggest that adjusting AHM regimens (preferring Ang-II stimulating AHMs, ahead of Ang-II inhibiting AHMs) might serve as a cost-effective preventive measure against dementia. Future research should explore the mechanisms behind these differences and investigate the effects of Ang-II stimulating AHM classes in diverse populations. Additionally, RCTs focusing on the impact of drug dose and treatment duration are needed to confirm these findings and fully understand the potential of these medications in dementia prevention.

## Supplementary Information

Below is the link to the electronic supplementary material.

Supplementary file1 (DOCX 2291 KB)

## Data Availability

All data are accessible within the manuscript or the supplementary files.
