# Supplementary material for: Comparative effects of angiotensin II stimulating and inhibiting antihypertensives on dementia risk: a systematic review and meta-analysis
Source: GeroScience. 2025 Apr 4;47(4):5525–41. doi: 10.1007/s11357-025-01600-1 (PMC12397011; doi:10.1007/s11357-025-01600-1)
Supplement: Supplementary file 1 — Supplementary file1 (DOCX 2291 KB) [file 11357_2025_1600_MOESM1_ESM.docx]

**Supplementary Tables**

Table S1. Search strategy for each database

| Pubmed | Concept 1 | Concept 2 | Concept 3 |
| --- | --- | --- | --- |
| Key concepts | Blood pressure and Hypertension | Antihypertensive medications | Dementia or Alzheimer's diseases or cognitive impairments |
| Free text terms / natural language terms | Hypertension [tiab]  OR  Blood pressure [tiab]  OR | Antihypertensive drugs [tiab] OR Antihypertensive agents [tiab] OR Antihypertensive Medications [tiab] OR Angiotensin II [Tiab] OR ARBs [Tiab] OR AT1 receptor blockers [Tiab] OR ACE inhibitors [Tiab] OR Angiotensin-converting enzyme blocker [Tiab] OR angiotensin-converting enzyme antagonists [Tiab] OR Angiotensin-converting enzyme inhibitor* [Tiab] OR Thiazide-type diuretics [Tiab] OR Thiazide-like diuretics [Tiab] OR Calcium antagonists [Tiab] OR Calcium entry blockers OR Calcium channel antagonist* OR Beta *blocker* [Tiab] OR adrenergic blocking agent [Tiab] OR | dement*[tiab]  OR  Cogni*[tiab]  OR  Alzheim*[tiab] |
| Controlled vocabulary terms / Subject terms  (MeSH terms, Emtree terms) | Hypertension [MeSH]  OR  Blood pressure [MeSH] | Antihypertensive agents [MeSH] OR angiotensin II [MeSH] OR Angiotensin Receptor Antagonists [MeSH] OR Angiotensin-converting enzyme inhibitors [MeSH] OR Diuretics [MeSH] OR Thiazides [MeSH] OR Calcium channel blockers [MeSH] OR adrenergic beta-antagonists [MeSH] | Dementia [MeSH Terms] |
| Search result | Search 1 result (#1) =906,401 | Search 2 result (#2) = 290,978 | Search 3 result (#3) =802,838 |
| Search result | #1 AND #2 AND #3=1754 | | |
| EMBASE Ovid | Concept 1 | Concept 2 | Concept 3 |
| Free text terms / natural language terms | Hypertension. ab,kf,ti.  OR  Blood pressure. ab,kf,ti.  OR | Antihypertensive drugs.ab,kf,ti. OR Antihypertensive agents.ab,kf,ti. OR Antihypertensive Medications.ab,kf,ti. OR Angiotensin II.ab,kf,ti. OR ARBs.ab,kf,ti. OR AT1 receptor blockers.ab,kf,ti. OR ACE inhibitors.ab,kf,ti. OR Angiotensin-converting enzyme blocker.ab,kf,ti. OR angiotensin-converting enzyme antagonists.ab,kf,ti. OR Angiotensin-converting enzyme inhibitor*.ab,kf,ti. OR Thiazide-type diuretics.ab,kf,ti. OR Thiazide-like diuretics.ab,kf,ti. OR Calcium antagonists.ab,kf,ti. OR Calcium entry blockers.ab,kf,ti. OR Calcium channel antagonist*.ab,kf,ti. OR Beta blocker* .ab,kf,ti. OR adrenergic blocking agent.ab,kf,ti. | dement*.ab,kf,ti.  OR  Alzheim*.ab,kf,ti.  OR  Cogni*.ab,kf,ti |
| Controlled vocabulary terms / Subject terms  (MeSH terms, Emtree terms) | Hypertension.sh.  OR  Blood pressure.sh | OR Antihypertensive agents .sh. ORangiotensin II.sh. OR angiotensin 1 receptor antagonist.sh. OR  Thiazides diuretics agent.sh. OR Calcium antagonist.sh.  OR adrenergic receptor blocking agent.sh. OR  dipeptidyl carboxypeptidase inhibitor.sh. | Dementia.sh. |
| Search result | Search 1 result (#1) =144465 | Search 2 result (#2) = 307893 | Search 3 result (#3) = 1077331 |
| Search result | #1 AND #2 AND #3=2277 | | |
| CINHAL | Concept 1 | Concept 2 | Concept 3 |
| Free text terms / natural language terms | (TI Hypertension OR  AB Hypertension   OR SU Hypertension) OR (TI "Blood pressure" OR AB "Blood pressure" OR SU "Blood pressure") OR | (TI "Antihypertensive drugs" OR AB "Antihypertensive drugs" OR SU "Antihypertensive drugs") OR (TI "Antihypertensive agents" OR AB "Antihypertensive agents" OR SU "Antihypertensive agents") OR (TI "Antihypertensive Medications" OR AB "Antihypertensive Medications" OR SU "Antihypertensive Medications") OR (TI "Angiotensin II" OR AB "Angiotensin II" OR SU "Angiotensin II") OR (TI ARBs OR AB ARBs OR SU ARBs) OR (TI "AT1 receptor blockers" OR AB "AT1 receptor blockers" OR SU "AT1 receptor blockers") OR (TI "ACE inhibitors" OR AB "ACE inhibitors" OR SU "ACE inhibitors") OR (TI "Angiotensin-converting enzyme blocker" OR AB "Angiotensin-converting enzyme blocker" OR SU "Angiotensin-converting enzyme blocker") OR (TI "angiotensin-converting enzyme antagonists" OR AB "angiotensin-converting enzyme antagonists" OR SU "angiotensin-converting enzyme antagonists") OR (TI "Angiotensin-converting enzyme inhibitor*" OR AB "Angiotensin-converting enzyme inhibitor*" OR SU "Angiotensin-converting enzyme inhibitor*") OR (TI "Thiazide-type diuretics" OR AB "Thiazide-type diuretics" OR SU "Thiazide-type diuretics") OR (TI "Thiazide-like diuretics" OR AB "Thiazide-like diuretics" OR SU "Thiazide-like diuretics") OR (TI "Calcium antagonists" OR AB "Calcium antagonists" OR SU "Calcium antagonists") OR (TI "Calcium entry blockers" OR AB "Calcium entry blockers" OR SU "Calcium entry blockers") OR(TI "Calcium channel antagonist*" OR AB "Calcium channel antagonist*" OR SU "Calcium channel antagonist*") OR (TI "Beta blocker*" OR AB "Beta blocker*" OR SU "Beta blocker*") OR (TI "adrenergic blocking agent" OR AB "adrenergic blocking agent" OR SU "adrenergic blocking agent")OR | (TI dement* OR AB dement* OR SU dement*) OR (TI Cogni* OR AB Cogni* OR SU Cogni*) OR (TI Alzheim* OR AB Alzheim* OR SU Alzheim*) OR (TI "Mini-Mental State Examination" OR AB "Mini-Mental State Examination" OR SU "Mini-Mental State Examination") OR |
| Controlled vocabulary terms / Subject terms  (MeSH terms, Emtree terms) | (MH Hypertension+) OR (MH "Blood pressure+") | (MH "Antihypertensive agents+") OR (MH "angiotensin II+") OR (MH "Angiotensin Receptor Antagonists+") OR (MH "Angiotensin-converting enzyme inhibitors+") OR (MH Diuretics+) OR (MH Thiazides+) OR (MH "Calcium channel blockers+") OR (MH "adrenergic beta-antagonists+") | (MH Dementia+)  OR (MH "Mini-Mental State Examination+") |
| Search result | Search 1 result (#1) =213,133 | Search 2 result (#2) = 61,816 | Search 3 result (#3) =312,155 |
| Search result | #1 AND #2 AND #3=709 | | |
| SCOPUS | Concept 1 | Concept 2 | Concept 3 |
| Free text terms / natural language terms | TITLE-ABS-KEY (Hypertension)  OR  TITLE-ABS-KEY (Blood pressure)  OR | TITLE-ABS-KEY (Antihypertensive drugs) OR TITLE-ABS-KEY (Antihypertensive agents) OR TITLE-ABS-KEY( Antihypertensive Medications) OR TITLE-ABS-KEY (Angiotensin II) OR TITLE-ABS-KEY (ARBs) OR (TITLE-ABS-KEY)AT1 receptor blockers OR TITLE-ABS-KEY (ACE inhibitors) OR TITLE-ABS-KEY (Angiotensin-converting enzyme blocker) OR TITLE-ABS-KEY (angiotensin-converting enzyme antagonists) OR TITLE-ABS-KEY (Angiotensin-converting enzyme inhibitor*) OR TITLE-ABS-KEY (Thiazide-type diuretics) OR TITLE-ABS-KEY (Thiazide-like diuretics) OR TITLE-ABS-KEY (Calcium antagonists) OR TITLE-ABS-KEY (Calcium entry blockers) OR TITLE-ABS-KEY (Calcium channel antagonist*)  OR TITLE-ABS-KEY (Beta blocker*) OR TITLE-ABS-KEY (adrenergic blocking agent) OR | TITLE-ABS-KEY (dement*) OR TITLE-ABS-KEY(Cogni*) OR TITLE-ABS-KEY(Alzheim*) OR TITLE-ABS-KEY ("Mini-Mental State Examination") OR |
| Controlled vocabulary terms / Subject terms  (MeSH terms, Emtree terms) | INDEXTERMS (Hypertension)  OR  INDEXTERMS (Blood pressure) | INDEXTERMS (Antihypertensive agents) OR INDEXTERMS (angiotensin II) OR INDEXTERMS (angiotensin 1 receptor antagonist) OR INDEXTERMS (Thiazides diuretics agent)  OR INDEXTERMS (Calcium antagonist) OR INDEXTERMS (adrenergic receptor blocking agent) OR INDEXTERMS (dipeptidyl carboxypeptidase inhibitor) | INDEXTERMS(Dementia) OR   INDEXTERMS ("Mini-Mental State Examination") |
| Search result | Search 1 result (#1) = **1,633,866** | Search 2 result (#2) =13,504 | Search 3 result (#3) =1,557,557 |
| Search result | #1 AND #2 AND #3= 148 | | |
| Pyscinfo ovid | Concept 1 | Concept 2 | Concept 3 |
| Free text terms / natural language terms | Hypertension. ab,ti.  OR  Blood pressure. ab,ti.  OR | Antihypertensive drugs.ab,ti. OR Antihypertensive agents.ab,ti. OR Antihypertensive Medications.ab,ti. OR Angiotensin II.ab,ti. OR ARBs.ab,ti. OR AT1 receptor blockers.ab,ti. OR ACE inhibitors.ab,ti. OR Angiotensin-converting enzyme blocker.ab,ti. OR angiotensin-converting enzyme antagonists.ab,ti. OR  Angiotensin-converting enzyme inhibitor*.ab,ti. OR Thiazide-type diuretics.ab,ti. OR Thiazide-like diuretics.ab,ti. OR Calcium antagonists.ab,ti. OR Calcium entry blockers.ab,ti. OR Calcium channel antagonist*.ab,ti. OR Beta blocker* .ab,ti.  OR adrenergic blocking agent.ab,ti. OR | dement*.ab,ti.  OR  Cogni*. ab ,ti.  OR Alzheim*.ab,ti.  OR Mini-Mental State Examination.ab,ti.OR |
| Controlled vocabulary terms / Subject terms  (MeSH terms, Emtree terms) | exp Hypertension/ or Hypertension.mp.  OR  Blood pressure.mp. or exp Blood Pressure/ | dipeptidyl carboxypeptidase inhibitor.mp OR exp Angiotensin/ or angiotensin II.mp. OR exp Antihypertensive Drugs/ or Antihypertensive agents.mp. OR angiotensin 1 receptor antagonist.mp. OR Thiazides diuretics agent.mp. OR exp Adrenergic Blocking Drugs/ or adrenergic receptor blocking agent.mp. OR exp Calcium Channel/ or Calcium antagonist.mp. | Dementia.mp. or exp Dementia/ ORMini-Mental State Examination.mp. or exp Mini Mental State Examination/ |
| Search result | Search 1 result (#1) = 41953 | Search 2 result (#2) = 29651 | Search 3 result (#3) =644994 |
| Search result | #1 AND #2 AND #3=421 | | |

Table S2: Baseline Patient Characteristics, Comorbidities, and Medication History

| Author and year | Baseline comorbidities and medication use history | | | | | | |
| --- | --- | --- | --- | --- | --- | --- | --- |
|  | SBP/DBP Mean (SD) | CAD/IHD | CHF | Stroke | DM | Use of statin | APOE4 positive |
| Barthold et al. 2018(1) | NR | 74093(6.5) | NR | 173624 (17) | 483826(48) | NR | NR |
| Cohen et al. 2022(2) | 143.5 (15.5/80.0 (12.3) | 277 (12.9) | NR | NR | NR | 491(32.95) | NR |
| Colbourne et al. 2022(3) | 137/77 | NR | NR | NR | NR | NR | NR |
| Diener et al.2008 (4) | NR | NR | NR | NR | NR | NR | NR |
| Du et al. 2023(5) | NR | NR | NR | NR | NR | NR | NR |
| Goh et al. 2014(6) | NR | NR | 35 353 (7.5) | 29 863 (7.0) | 110739 (23.5)) | 193 360 (41.2) | NR |
| Hu et al.2020(7) | 156.73 (9.94)/ 70.80 (7.83) | NR | NR | NR | NR | NR | 164(26.3) |
| Hwang et al. 2016 (8) | 141.3(18.62)/84.33 (11.49) | 2,896 (21.2) | NR | NR | 2,554 (18.7) | NR | NR |
| Li et al. 2010 (9) | 135 (13)/73.5 (8) | 31104 (32) | NR | 13323(12.5) | 41804 (38) | NR | NR |
| Lithell et al.2004 (10) | 164.7 (9.1)/ 90.4 (6.2) | 83 (4.0) | NR | 71 (3.4) | 236 (11.2) | NR | NR |
| Marcum et al.2022 (11) | 139.7 (14.6)/79.4 (11.5) | NR | NR | NR | NR | 1714 (43) | NR |
| Marcum et al.2023(12) | NR | 4006 (20.3) | 1390 (7.3) | 876 (4.7) | 2271(11.4) | 3983 (21) | NR |
| Schroevers et al.2023 (13) | 156.2(21.5)/81.4 (11.2) | 947 (49.7) | NR | NR | 501 (26.3) | NR | NR |
| Schroevers et al.2024 (14) | NR | 13,793 (10.3) | 5846 (4.4) | 9075 (6.8 | 26,655 (20.0) | NR | NR |
| Tzourio et al.2003(PROGRESS)(15) | NR | NR | NR | NR | NR | 64(10) | 563(22) |
| Van Dalen et al.2021(16) | 156.6/ 81.4 | 811(43) | NR | 257(13.7) | 504 (25.9) | 921 (48.2) | 446 (27.6) |
| Whiteley et al.2021(17) | 162 (18)/92 (10 | 1479 (17.2%) | NR | 999 (11.65) | 1455(28.6) | NR | NR |
| Yasar et al. 2005(18) | NR | 184 (82.7) | NR | NR | NR | NR | NR |

Abbreviations: CAD/IHD; Coronary Artery Disease/Ischemic Heart Disease, CHF; Congestive Heart Failure, DBP; Diastolic Blood Pressure, DM; Diabetes Mellitus, IQR; Interquartile Range, N (%); Number (Percentage), NR; Not Reported, SBP; Systolic Blood Pressure, SD; Standard Deviation

Table S3: Study characteristics

| Author and year | Aim of the study | Study population and inclusion criteria | Exclusion criteria | Definition of AHMs exposure | Outcome Definition | Events for secondary outcome, n (%) | | | | | | Outcome and Findings/HR/, 95%CI for second outcomes | Adjustments for confounders |
| --- | --- | --- | --- | --- | --- | --- | --- | --- | --- | --- | --- | --- | --- |
| Barthold et al. 2018 (1) | Examined differences in RAS-acting drugs on AD and variations by sex and race/ethnicity. | Medicare beneficiaries aged 67 and older use at least one AHMs for two consecutive years, no prior AD diagnoses, and no prior use of AChEI. | Previous diagnosis of dementia, previous AHMs use. | AHT user is any individual with 90 days’ supply and at least two drug claims in a year for two consecutive years. | ICD-9 | N/A | | | | | | N/A | Unadjusted |
| Cohen et al.2022(2) | Evaluate the cognitive outcomes of initiating an ARBs vs ACEIs. | Aged 50 years or older with an elevated risk of CVD SBP 130 to 180 mm Hg | DM, history of stroke, CHF, living in a nursing home, with a diagnosis of dementia or receiving medications for dementia | Use of AHMs for 1 year period | MoCA, WAIS | Ang^+^ | | PD:20 (0.70) | | | | PD alone: HR (ref. Ang^-^)  Ang^+^: 1.02 (0.58-1.79) | Unadjusted |
|  |  |  |  |  |  | Ang^-^ | | PD:41 (0.70) | | | |  |  |
| Colbourne et al. 2022 (3) | Investigate if BBB-CCBs are linked to a lower incidence of psychiatric and neurodegenerative disorders compared to amlodipine. | Aged 18–90 years old at index, free of any history of “organic” psychiatric disorder including dementia and delirium, and had no prior exposure to the AHMs | Prior psychiatric diagnosis | Prescriptions of AHMs for at least two years | ICD-10 | N/A | | | | | | N/A | Unadjusted |
| Diener et al.2008 (PRoFESS)(4) | Neuroprotective effects of antiplatelet compounds and the angiotensin II receptor antagonist telmisartan in the PRoFESS trial | Patients aged 55 years and above,  and stroke<90 days; age 50-54 year or stroke 90-120 if ≥2CVRF | ICH/SAH, brain tumour, dementia, CKD, CAD, SBP ≥180, DBP ≥110 | Use of AHMs throughout the follow-up time. | MMSE ≤ 24 or **≥** 3points decline and clinical impression | N/A | | | | | | N/A | Unadjusted |
| Du et al, 2023(5) | Assess the risk of AD and ADRD from antihypertensive medications in colorectal cancer survivors | Patients who were diagnosed with colorectal cancer at age ≥65 years and HTN | Patients without Parts A and B enrolled in HMO or Part C or died within 30 days of cancer diagnosis. | MPR ≥80% | ICD-9 and ICD -10 | Ang^+^ | AD: 341(4.3) | | | | | HR adjusted (ref. Ang^-^)  Ang^±^: 1.18 (1.13-1.24) *  AD: HR adjusted (ref. Ang^-^)  Ang^+^: 0.87 (0.76–0.99)  Ang^±^: 1.43 (1.28-1.59) *  VD: HR adjusted (ref. Ang^-^)  Ang^+^: 0.78 (0.65-0.95)  Ang^±^: 1.32 (1.14-1.53) *  MCI: HR adjusted (ref. Ang^-^)  Ang^+^:0.79 (0.63-0.98)  Ang^±^: 1.23 (1.03-1.47) * | Ages, gender, ethnicity, marital status, tumour stage, grade, site, chemotherapy, comorbidity score, year of diagnosis |
|  |  |  |  |  |  |  | VD: 167 (2.1) | | | | |  |  |
|  |  |  |  |  |  |  | MCI: 119 (1.5) | | | | |  |  |
|  |  |  |  |  |  | Ang^-^ | AD: 590 (4.2) | | | | |  |  |
|  |  |  |  |  |  |  | VD: 337 (2.4) | | | | |  |  |
|  |  |  |  |  |  |  | MCI: 239 (1.7) | | | | |  |  |
|  |  |  |  |  |  | Ang^±^ | AD: 701 (6.1) | | | | |  |  |
|  |  |  |  |  |  |  | VD: 368 (3.2) | | | | |  |  |
|  |  |  |  |  |  |  | MCI: 241 (2.1) | | | | |  |  |
| Goh et al.2014(6) | Evaluate if ARBs lower dementia risk compared to ACEIs. | All patients aged ≥18 years with a first recorded prescription for an ACEI or ARB | Prevalent antihypertensive user  Dementia at baseline | Ever use of AHMs during follow-up based on CPRD data | Specific codes in UKCPRD | N/A | | | | | | N/A | Age, sex, BMI, DM, BP, CHF, statin use, socioeconomic status, alcohol, smoking, number of consultations, calendar year. |
| Hu et al.2020(7) | Study the impact of telmisartan, rosuvastatin, or their combination on dementia and the role of APOE genotype | Essential hypertensive patients, aged ≥60 year | AD, Parkinson’s, schizophrenia, seizures, MMSE ≤23, secondary hypertension, DM, recent MI or stroke, hypersensitivity to study meds, chronic liver or renal disease, inflammatory muscle disease, malignancy, drug/alcohol abuse, plans to move, inability to walk to clinic, unwillingness to consent. | Use of AHMs throughout the follow up time. | MMSE, MoCA, DRS, and CDR | N/A | | | | | | N/A | Unadjusted |
| Hwang et al. 2016(8) | Investigate the protective effects of CCBs on dementia. | Elderly hypertensive patients and age ≥60 years | Dementia, CVD, cancer | MPR≥80% | At least 1 dementia record as an admission or 3 records as an outpatient | Ang^+^ | | | | | AD: 1,206 (10.2) | HR adjusted (ref. Ang^-^)  AD; Ang^+^:0.88(0.60-1.28) *  VD; Ang^+^:1.00(0.55-1.81) * | Age, sex, BP, BMI, income, urbanization, smoking, alcohol consumption, CAD, hypercholesterolemia, DM, number of AHMs |
|  |  |  |  |  |  |  |  |  |  |  | VD: 536 (4.5) |  |  |
|  |  |  |  |  |  | Ang^-^ | | | | | AD: 28 (10.4) |  |  |
|  |  |  |  |  |  |  |  |  |  |  | VD: 11 (4) |  |  |
| Li et al. 2010 (9) | Investigate if angiotensin receptor blockers protect against or slow Alzheimer's and dementia. | Aged ≥65 with cardiovascular disease. | History of dementia | MPR≥80% | ICD-9 | Ang^+^ | | | | | AD: 140 (1.2) | AD: HR adjusted (ref. Ang^-^)  Ang^+^: 0.81(0.68 - 0.96) | Age, stroke, DM, cardiovascular disease |
|  |  |  |  |  |  | Ang^-^ | | | | | AD: 1,344 (1.44) |  |  |
| Lithell et al. 2004(10) | Assess candesartan (8–16 mg daily) effects in elderly patients (70–89 years) with mild to moderate hypertension. | Aged 70–89 years with SBP 160– 179 mmHg and/or DBP 90–99 mmHg, reserved cognitive function | Dementia, AHMs other than HCTZ, SecHTN, stroke/MI, <6 month, HDF, CKD | AHM use during follow-up. | Modified ICD-10 | N/A | | | | | | N/A | Unadjusted |
| Marcum et al. 2022(11) | Examine how antihypertensives that stimulate vs. inhibit type 2 and 4 angiotensin II receptors affect MCI or dementia. | Individuals 50 years or older with hypertension and increased cardiovascular risk | NR | Use of AHMs for at least six-month period | MoCA, WAIS, WMS | Ang^+^ | | | PD:73 (2.76) | | | HR adjusted (ref. Ang^-^)  PD:HR (ref. Ang^-^)  Ang^+^:0.80 (0.57-1.14) | Unadjusted |
|  |  |  |  |  |  | Ang^-^ | | | PD:67 (4.3) | | |  |  |
| Marcum et al, 2023(12) | Examine the link between new antihypertensives (stimulate vs. inhibit type 2 and 4 angiotensin II receptors) and ADRD | Medicare fee-for-service beneficiaries aged 65 years or older with incident hypertension | History of HTN, Patients taking AHMs before the index date, dementia, age</65 years | MPR≥80% | ICD-9 and ICD -10 | Ang^+^ | | | VD: 513 (4.9) | | | VD:HR adjusted (ref. Ang^-^_)_  Ang^+^: 0.82 (0.69-0.96)  Ang^±^:0.83 (0.70-0.98) | Age, sex, race, ethnicity, income, atrial fibrillation, IHD, DM, CHF, depression,  CKD, and stroke |
|  |  |  |  |  |  | Ang^-^ | | | VD: 513 (4.9) | | |  |  |
|  |  |  |  |  |  | Ang^±^ | | | VD: 179 (8.2) | | |  |  |
| Schroevers et al.2023(13) | Investigate the impact of AHMs on dementia and whether effects are sustained long-term. | Community-dwelling older adults without prior diagnosis of dementia | Patients not used AHMs at baseline | Baseline AHM use | DSM-IV | N/A | | | | | | N/A | Age, sex, CVD, DM |
| Schroevers et al.2024(14) | Investigate AHM (sub-)classes and dementia risk using large-scale real-world prescription and outcome data. | Aged ≥65 years and use AHMs | NR | ≥3successive prescriptions of an AHM-class | ICPC | N/A | | | | | | N/A | Age, sex, DM, MI, stroke, K-sparing & Loop diuretics |
| Tzourio et al.2003(PROGRESS)(15) | Assess if lowering blood pressure reduces dementia or cognitive decline risk in CVD patients. | People with prior stroke or transient ischemic attack | Compelling (contra)indication for ACEi, SAH | Use throughout the follow up period | DSM-IV | N/A | | | | | | N/A | Unadjusted |
| Van Dalen et al.2021(16) | Compare incident dementia risk between angiotensin II–stimulating and inhibiting antihypertensives. | Community dwelling older (age 70-78 years) | Dementia, terminal illness, alcoholism | Baseline AHMs use | DSM-IV | N/A | | | | | | N/A | BP, DM, Stroke, CVD, BMI, LDL, APOE4 positive, education |
| Whiteley et al.2021(17) | Assess if dementia or stroke are linked to different BP-lowering regimens | Participants with hypertension and ≥3CVRF | NR | Use of AHMs throughout the follow up time. | ICD-10 | Ang^+^ | | | | AD: 105 (2.44) | | AD: HR adjusted (ref. Ang^-^)  Ang^+^: 0.90 (0.73-1.15)  VD: HR adjusted (ref. Ang^-^)  Ang^+^: 0.92 (0.73-1.15) | Unadjusted |
|  |  |  |  |  |  |  |  |  |  | VD:141 (3.28) | |  |  |
|  |  |  |  |  |  | Ang^-^ | | | | AD:116 (2.71) | |  |  |
|  |  |  |  |  |  |  |  |  |  | VD: 153 (3.58) | |  |  |
| Yasar et al. 2005(18) | Investigate the link between CCBs (diCCBs vs. non-diCCBs) and risk of Alzheimer’s or mortality. | Subjects are volunteers recruited from the Baltimore-Washington area andage≥60 years at last follow up | History of dementia | Use of AHMs at some time during follow-up | DSM-III-R, NINCDS-ADRDA | N/A | | | | | | N/A | Age, sex, education, income, number of  vascular diseases, BMI, SBP and DBP |

Abbreviations: Ang+, angiotensin II–stimulating medication; Ang-, angiotensin II–inhibiting medication; Ang±, both angiotensin II–stimulating and angiotensin II–inhibiting; AChEI, Acetylcholine Esterase Inhibitors **;** ACEIs, Angiotensin-Converting Enzyme Inhibitors; AD, Alzheimer's Disease; AHMs, Antihypertensive Medications; ARBs, Angiotensin II Receptor Blockers; CAD, Coronary Artery Disease; CCB, Calcium Channel Blockers; CKD, Chronic Kidney Disease; CPRD, Clinical Practice Research Datalink; CVRF, Cardiovascular Risk Factors; CVD, Cardiovascular Disease; CDR, Clinical Dementia Rating; CPRD, Clinical Practice Research Datalink; DBP, Diastolic Blood Pressure, DHP CCBs, Dihydropyridine Calcium Channel Blockers; DM, Diabetes Mellitus; DRS, Dementia Rating Scale; DSM-III/IV, Diagnostic and Statistical Manual of Mental Disorders, Three/Fourth Edition; HR, Hazard Ratio; HCTZ, Hydrochlorothiazide; HMO, Health Maintenance Organization; HDF, Heart Disease Factors; HTN, Hypertension; ICD-9/10, International Classification of Diseases, 9th /10^th^ Revision; ICPC, International Classification of Primary Care; MCI, Mild Cognitive Impairment; MMSE; MI, Myocardial Infarction; Mini-Mental State Examination; MPR, Medication Possession Ratio; MoCA, Montreal Cognitive Assessment; n (%), Number (Percentage),  N/A, Not Assessed; NR, Not Reported; NINCDS-ADRDA, National Institute of Neurological and Communicative Disorders and Stroke and the Alzheimer's Disease and Related Disorders Association; PD, Probable dementia; RAS, Renin-Angiotensin System; SBP, Systolic Blood Pressure,; SecHTN, Secondary Hypertension, UKCPRD, UK Clinical Practice Research Datalink; VD, Vascular Dementia; WAIS, Wechsler Adult Intelligence Scale; WMS, Wechsler Memory Scale

Table S4: Assessment of the included Randomised Controlled Trials (RCTs) using Joanna Briggs Institute (JBI) critical appraisal tools.

| Author and year | JBI Critical Appraisal Checklists for RCTs (Response: Yes, No, Unclear or Not applicable) | | | | | | | | | | | | | Total score | Study quality |
| --- | --- | --- | --- | --- | --- | --- | --- | --- | --- | --- | --- | --- | --- | --- | --- |
|  | Q1 | Q2 | Q3 | Q4 | Q5 | Q6 | Q7 | Q8 | Q9 | Q10 | Q11 | Q12 | Q13 |  |  |
| Diener et al.2008 (4) | Y | Y | Y | Y | Y | Y | Y | Y | U | Y | Y | Y | Y | 92.3% | High |
| Hu et al.2020 (7) | Y | Y | Y | Y | Y | Y | Y | Y | Y | U | Y | Y | Y | 92.3% | High |
| Lithell et al .2004 (10) | Y | Y | Y | Y | Y | Y | Y | Y | Y | N | Y | Y | Y | 92.3% | High |
| Tzourio et al.2003 (15) | Y | Y | Y | Y | Y | Y | Y | Y | Y | Y | Y | Y | Y | 100% | High |
| Whiteley et al.2021(ASCOT) (17) | Y | Y | Y | N | N | U | N | Y | Y | N | Y | Y | Y | 50% | Moderate |

Scores can range from 0 to 13, each question is given a single score for yes and 0 for no, unclear, and not applicable (NA); High quality: % of score >70, Moderate quality: % of score 50-70, Low quality: % of score <50

Q1: Was true randomisation used for the assignment of participants to treatment groups? Q2: Was allocation to treatment groups concealed? Q3: Were treatment groups similar at the baseline? Q4: Were participants blind to treatment assignment? Q5: Were those delivering the treatment blind to the treatment assignment? Q6: Were treatment groups treated identically other than the intervention of interest? Q7: Were outcome assessors blind to treatment assignment? Q8: Were outcomes measured in the same way for treatment groups? Q9: Were outcomes measured in a reliable way? Q10: Was follow-up complete, and if not, were differences between groups in terms of their follow-up adequately described and analysed? Q11: Were participants analysed in the groups to which they were randomised? Q12: Was appropriate statistical analysis used? Q13: Was the trial design appropriate, and any deviations from the standard RCT design (individual randomisation, parallel groups) accounted for in the conduct and analysis of the trial?

Table S5: Assessment of cohort studies using Joanna Briggs Institute (JBI) critical appraisal tools.

| Author year | JBI Critical Appraisal Checklists for cohort studies (Response: Yes, No, Unclear or Not applicable) | | | | | | | | | | | | Total score | Study quality |
| --- | --- | --- | --- | --- | --- | --- | --- | --- | --- | --- | --- | --- | --- | --- |
|  | Q1 | Q2 | Q3 | Q4 | Q5 | Q6 | Q7 | Q8 | Q9 | Q10 | Q11 |  | |  |
| Barthold et al.2018 (1) | Y | Y | Y | Y | Y | Y | Y | Y | U | Y | Y | 90.9% | | High |
| Cohen et al .2022 (2) | Y | Y | Y | Y | Y | Y | Y | Y | Y | Y | Y | 100% | | High |
| Colbourne et al. 2022 (3) | Y | Y | Y | Y | Y | Y | Y | N | U | NA | Y | 72.7% | | High |
| Du et al. 2023 (5) | Y | Y | Y | Y | Y | Y | Y | Y | U | Y | Y | 90.9% | | High |
| Goh et al .2014 (6) | Y | Y | Y | Y | Y | Y | U | Y | Y | Y | Y | 90.9% | | High |
| Hwang et al. 2016 (8) | Y | Y | Y | Y | Y | Y | Y | Y | N | N | Y | 81.8% | | High |
| Li et al. 2010 (9) | Y | Y | Y | Y | Y | Y | Y | Y | Y | N | Y | 90.90% | | High |
| Marcum et al. 2022 (11) | Y | Y | Y | Y | Y | Y | Y | Y | U | Y | Y | 90.90% | | High |
| Marcum et al. 2023 (12) | Y | Y | Y | Y | Y | Y | Y | Y | Y | Y | Y | 90.9% | | High |
| Schroeder’s et al.2023 (13) | Y | Y | Y | Y | Y | Y | N | Y | Y | NA | Y | 81.8% | | High |
| Schroeder’s et al.2024 (14) | Y | Y | Y | Y | Y | Y | Y | Y | NA | Y | Y | 90.9% | | High |
| Van Dalen et al.2021 (16) | Y | Y | Y | Y | Y | Y | Y | Y | N | Y | Y | 90.90% | | High |
| Yasar et al. 2005 (18) | Y | Y | Y | Y | Y | Y | Y | Y | Y | Y | Y | 100% | | High |

Scores can range from 0 to 11; each question is given a single score for yes and 0 for no, unclear, and not applicable (NA); High quality: % of score >70, Moderate quality: % of score 50-70, Low quality: % of score <50

Q1: Were the two groups similar and recruited from the same population? Q2: Were the exposures measured similarly to assign people to both exposed and unexposed groups? Q3: Was the exposure measured in a valid and reliable way? Q4: Were confounding factors identified? Q5: Were strategies to deal with confounding factors stated? Q6: Were the groups/participants free of the outcome at the start of the study (or now of exposure)? Q7: Were the outcomes measured in a valid and reliable way? Q8: Was the follow-up time reported sufficient to be long enough for outcomes to occur? Q9: Was the follow-up complete, and if not, were the reasons for loss to follow-up described and explored? Q10: Were strategies to address incomplete follow-up utilised? Q11: Was appropriate statistical analysis use

Table S6. Sensitivity analysis for Heterogeneity using the leave-one-out method

| Omitted study | I^2^ | Chi² | P |
| --- | --- | --- | --- |
| Colbourne et al. 2022 | 40.21 | 17.12 | 0.0718 |
| Du et al.2023 | 59.84 | 19.66 | .0327 |
| Goh et al. 2014 | 65.93 | 23.48 | 0.0091 |
| Hwang et al. 2016 | 68.54 | 25.06 | 0.0052 |
| Li et al. 2010 | 66.51 | 23.22 | 0.0099 |
| Marcum et al.2023 | 70.07 | 24.53 | 0.0063 |
| Schroevers et al.2024 | 70.40 | 25.15 | 0.0051 |
| Van Dalen et al.202 | 66.31 | 22.29 | 0.0137 |

Table S7: Subgroup Analysis of Ang-II Stimulating vs. Inhibiting AHMs for Dementia Prevention

| **Analysis Group** | **No of Studies (references)** | **Pooled hazard ratio**  **(95% CI)** | **P-value** | **Tests for Heterogenicity** | |
| --- | --- | --- | --- | --- | --- |
|  |  |  |  | **P Value**  **(Q Statistic)** | **I^2^(%)** |
| Age  ≥71years  <71 years | (n = 3)(5, 9, 16)  (n = 5) (3, 6, 8, 12, 14) | 0.81 (0.78-0.86)  0.91(0.85-0.97) | P < 0.01  P < 0.01 | P=0.35  P=0.09 | 0.00%  55.58 |
| Sex: % Female  ≥55%  < 55% | (n = 5) (3, 5, 8, 12, 14)  (n = 3) (6, 9, 16) | 0.88 (0.81-0.96)  0.84 (0.73-0.97) | P < 0.01  P < 0.01 | P=0.01  P=0.04 | 73.96  69.0% |
| Study design  Retrospective cohort  Prospective cohort | (n = 3) (5, 12, 14)  (n = 5) (3, 6, 8, 9, 16) | 0.84 (0.80-0.88)  0.89 (0.80-1.00) | P < 0.01  P=0.05 | P=0.32  P=0.01 | 21.68%  68.32% |
| Country  USA  Europe | (n = 3) (5, 9, 12)  (n = 4) (3, 6, 14, 16) | 0.82 (0.79-0.86)  0.92 (0.84-1.00) | P < 0.01  P=0.05 | P=0.86  P=0.03 | 0.01%  57.88% |
| Diabetes Meletus  ≥22% DM  <22% DM | (n = 3) (6, 9, 16)  (n = 3) (8, 12, 14) | 0.84 (0.77-0.92)  0.88 (0.84-0.92) | P < 0.01  P < 0.01 | P=0.07  P=0.53 | 54.17  0.01 |
| Stroke  ≥7% stroke  <7% stroke | (n = 3) (1, 9, 16)  (n = 2) (12, 14) | 0.84 (0.77-0.92)  0.88 (0.84-0.92) | P < 0.01  P < 0.01 | P=0.07  P=0.34 | 54.17  0.01 |
| CAD/IHD  ≥21.2%  <21.2% | (n = 3) (8, 9, 16)  (N = 2)(12, 14) | 0.81 (0.74-0.89)  0.88 (0.86-0.90) | P < 0.01  P < 0.01 | P=0.40  P=0.62 | 0.00  0.09 |

**Supplementary Figures**


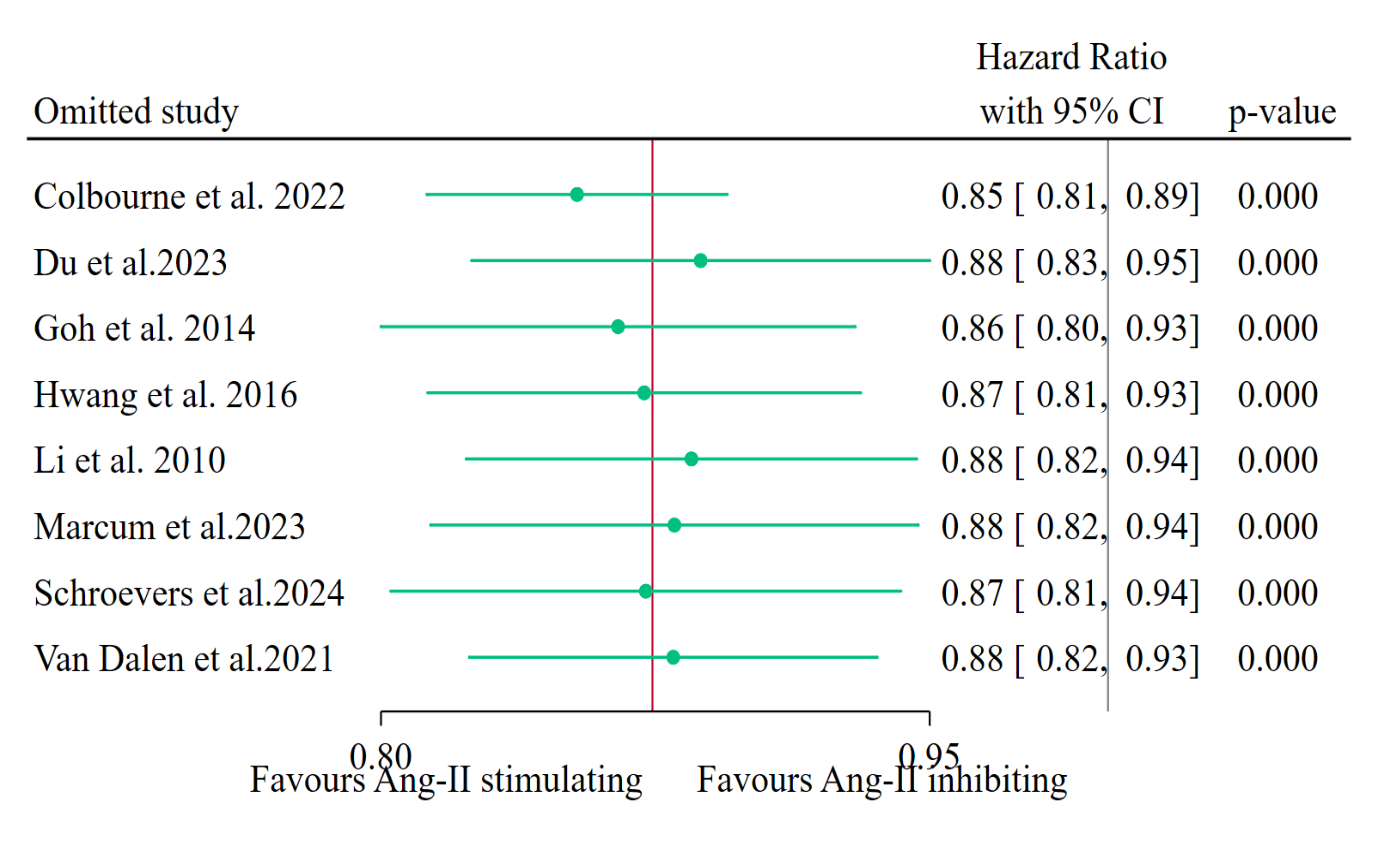


Figure S1. Sensitivity analysis via the leave-one-out method evaluates the impact of Ang-II AHMs on the incidence of all-cause dementia in comparison to Ang-II inhibitors. Each study was sequentially excluded using a random effects model.


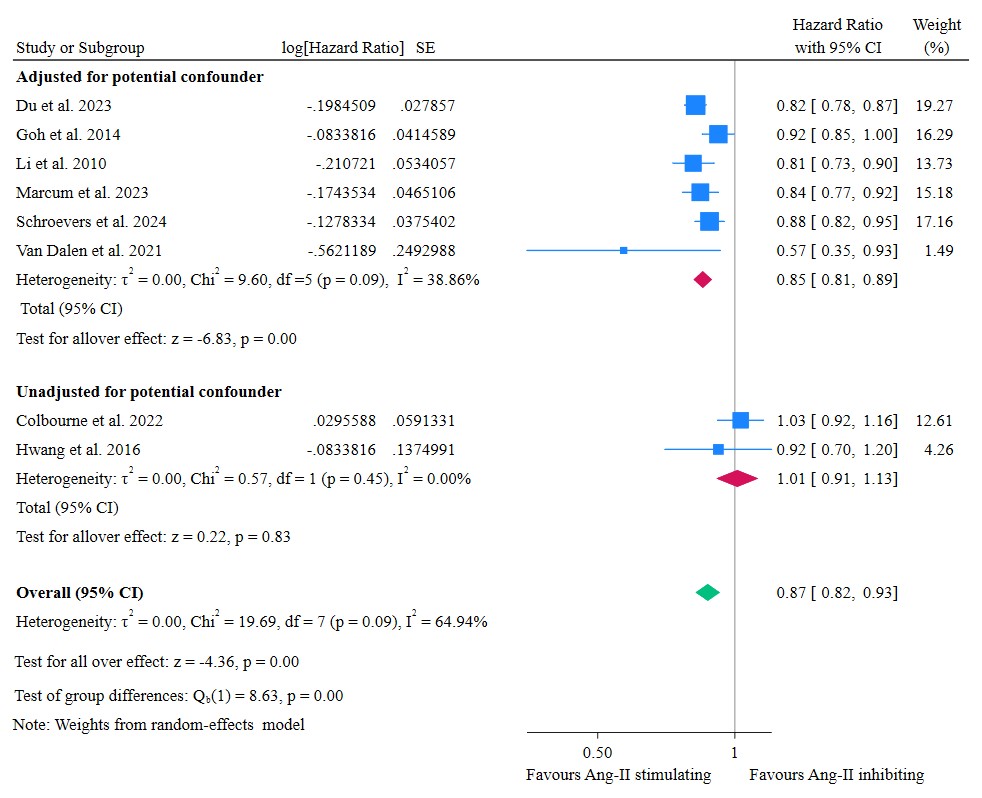


Figure S2: Forest plot of the incident all-cause dementia between angiotensin-II stimulating and inhibiting antihypertensive medication subgroup analysis by adjustment for confounders


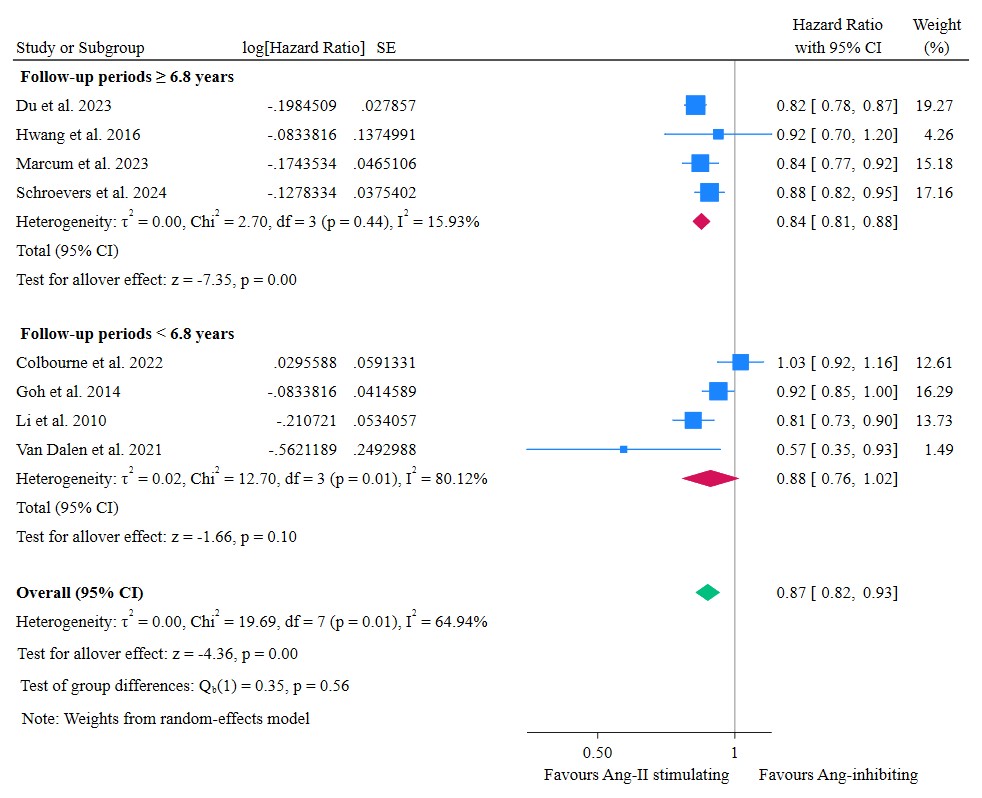


Figure S3: Forest plot of the incident dementia between angiotensin-II stimulating and inhibiting antihypertensive medication subgroup analysis by duration of follow-up


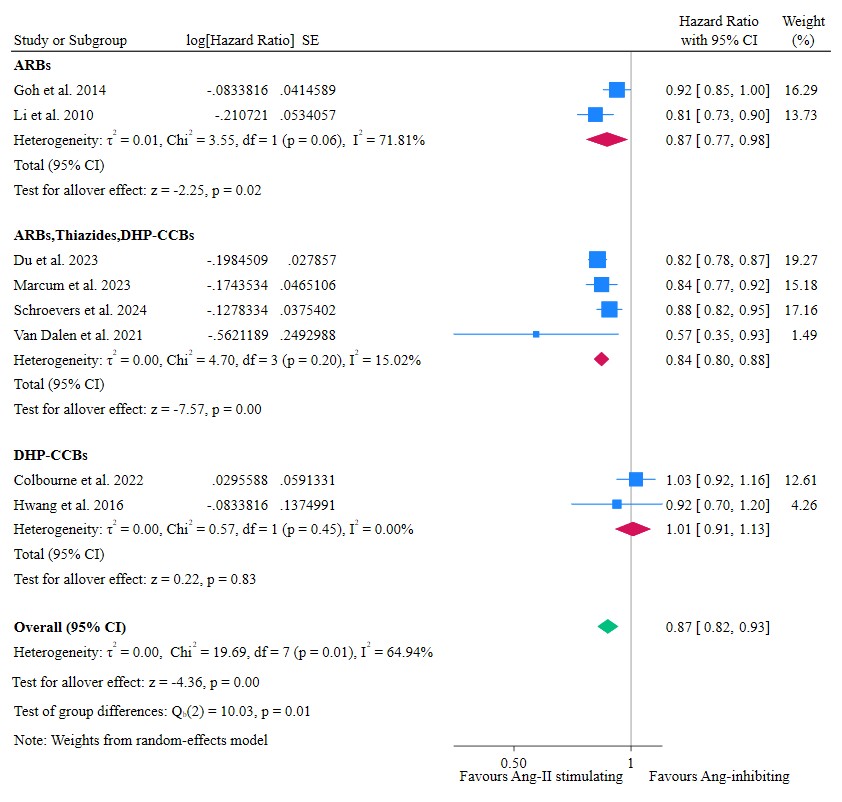


Figure S4. Forest plot of the incident dementia between angiotensin-II stimulating and inhibiting antihypertensive medication subgroup analyses by exposure class of AHMs


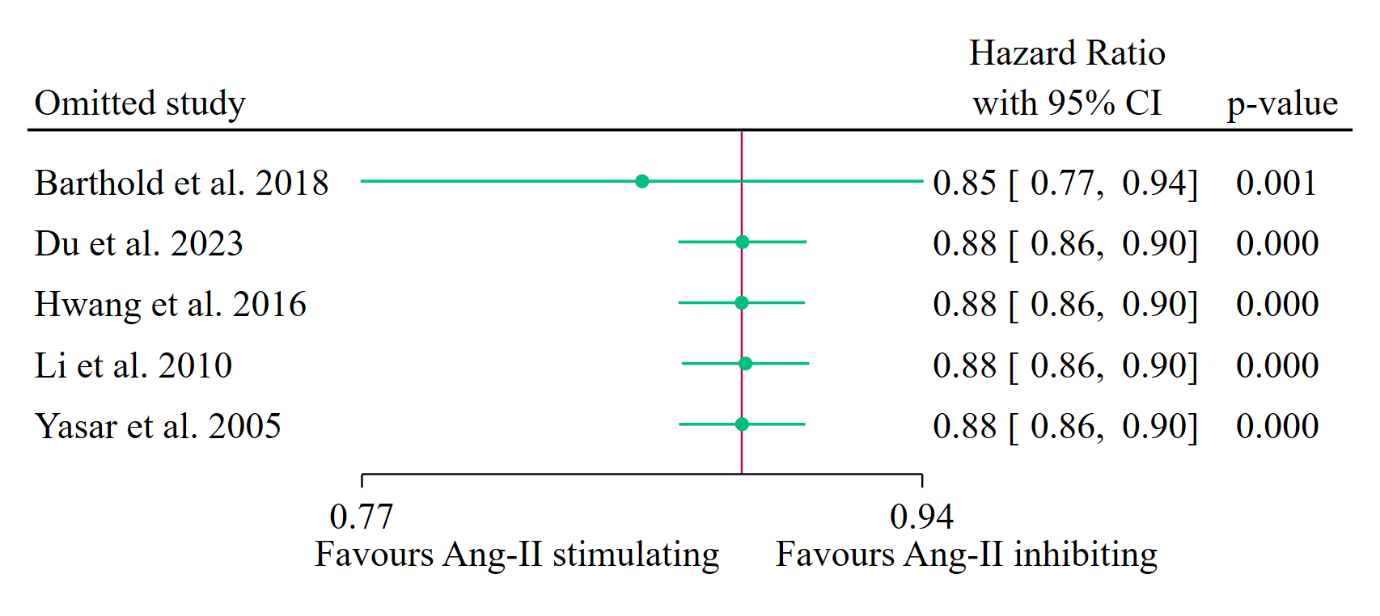


Figure S5. Sensitivity analysis via the leave-one-out method evaluates the impact of Ang-II AHMs on the incidence of Alzheimer’s disease in comparison to Ang-II inhibitors. Each study was sequentially excluded using a random effects model.

Supplementary S6. Publication bias using funnel plot


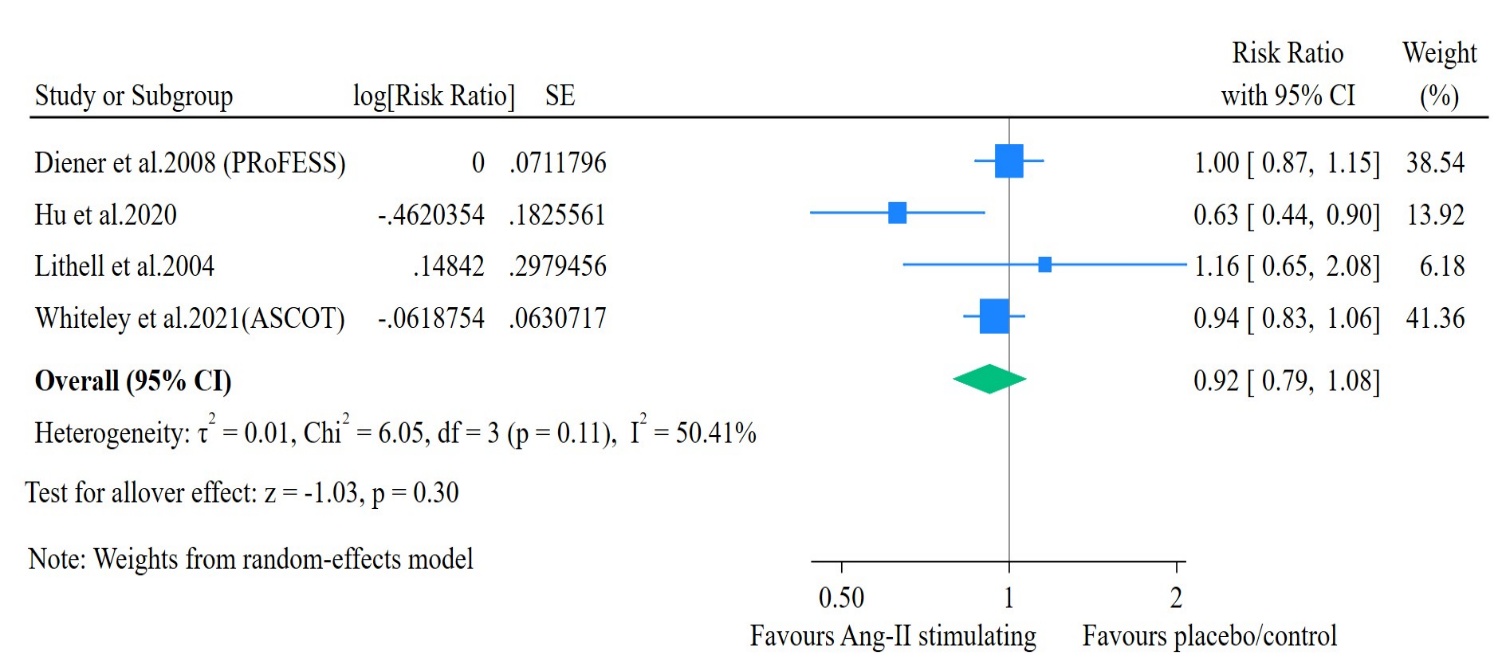


Figure S7: Incidence of all-cause dementia between Ang-II stimulating antihypertensive medication and placebo/control users.


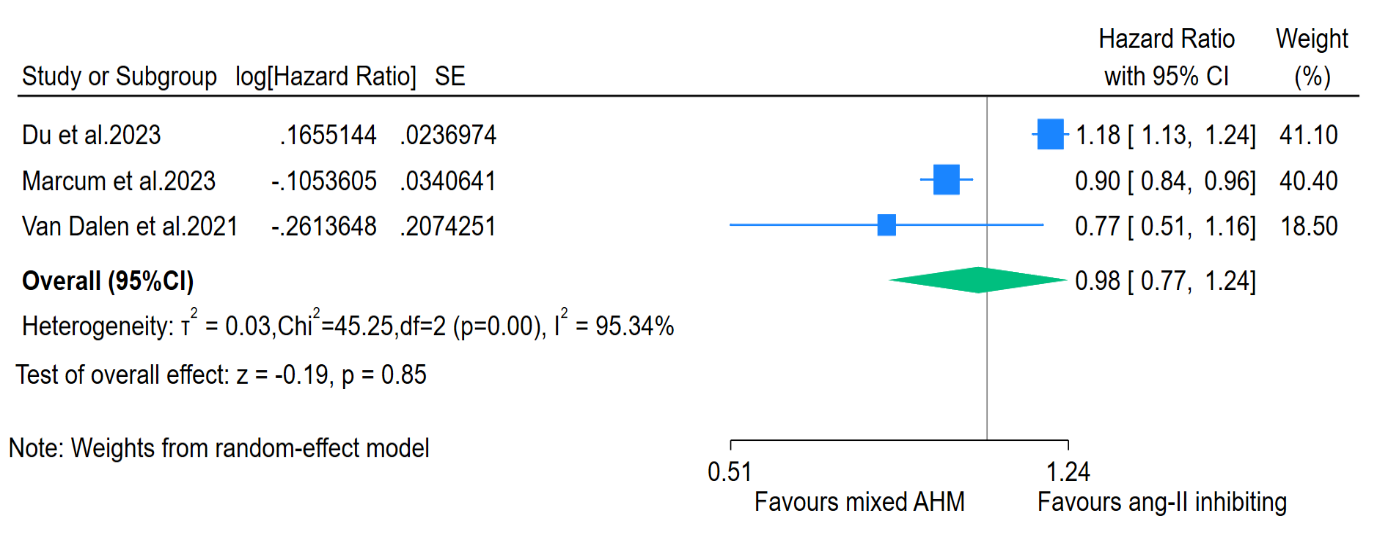


Figure S8: Incidence of all-cause dementia between mixed antihypertensive medication and Ang-II inhibiting user group.


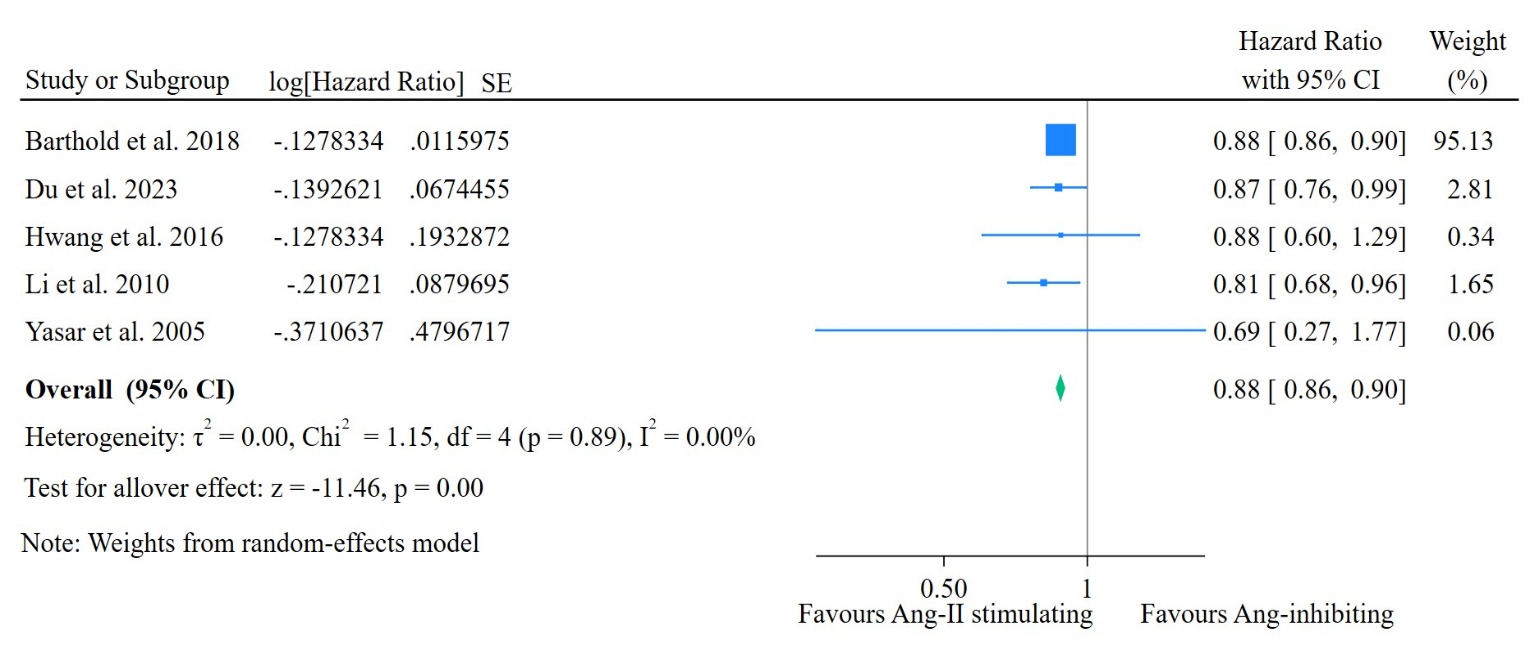


Figure S9: Incidence of Alzheimer’s disease between Ang-II stimulating and Ang-II inhibiting user group


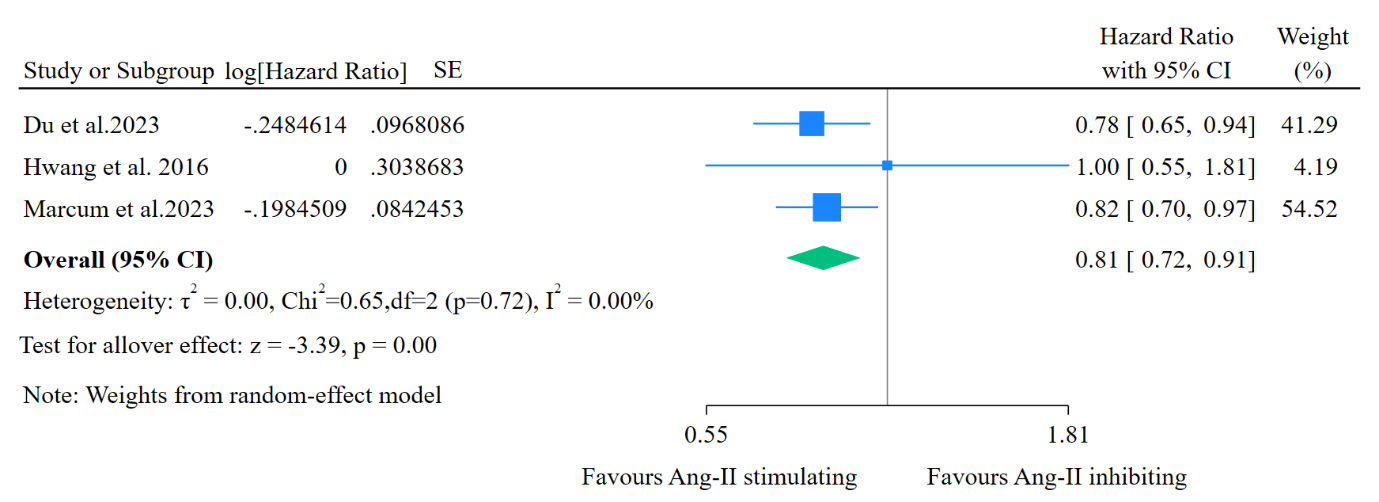


Figure 10: Incidence of vascular dementia between Ang-II stimulating and Ang-II inhibiting user groups.


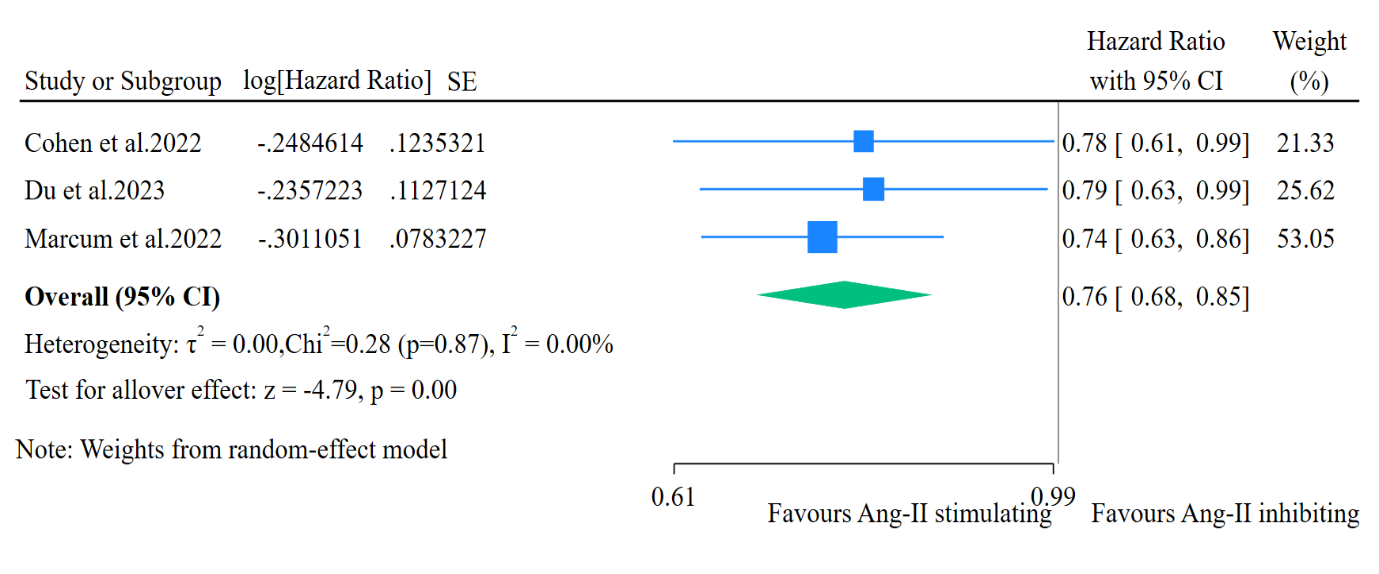
Figure S11: Incidence of mild cognitive impairment between Ang-II stimulating and Ang-II inhibiting user group.

**Reference**

1. Barthold D, Joyce G, Wharton W, Kehoe P, Zissimopoulos J. The association of multiple anti-hypertensive medication classes with Alzheimer’s disease incidence across sex, race, and ethnicity. PloS one. 2018;13(11):e0206705.

2. Cohen JB, Marcum ZA, Zhang C, Derington CG, Greene TH, Ghazi L, et al. Risk of mild cognitive impairment or probable dementia in new users of angiotensin II receptor blockers and angiotensin-converting enzyme inhibitors: a secondary analysis of data from the Systolic Blood Pressure Intervention Trial (SPRINT). JAMA Network Open. 2022;5(7):e2220680-e.

3. Colbourne L, Harrison PJ. Brain-penetrant calcium channel blockers are associated with a reduced incidence of neuropsychiatric disorders. Mol Psychiatry. 2022;27(9):3904-12.

4. Diener HC, Sacco RL, Yusuf S, Cotton D, Ounpuu S, Lawton WA, et al. Effects of aspirin plus extended-release dipyridamole versus clopidogrel and telmisartan on disability and cognitive function after recurrent stroke in patients with ischaemic stroke in the Prevention Regimen for Effectively Avoiding Second Strokes (PRoFESS) trial: a double-blind, active and placebo-controlled study. Lancet Neurol. 2008;7(10):875-84.

5. Du XL, Li Z, Schulz PE. Angiotensin-II stimulating vs. inhibiting antihypertensive drugs and the risk of Alzheimer's disease or related dementia in a large cohort of older patients with colorectal cancer. Frontiers in Cardiovascular Medicine. 2023;10:1136475.

6. Goh KL, Bhaskaran K, Minassian C, Evans SJ, Smeeth L, Douglas IJ. Angiotensin receptor blockers and risk of dementia: cohort study in UK Clinical Practice Research Datalink. Br J Clin Pharmacol. 2015;79(2):337-50.

7. Hu W, Li Y, Zhao Y, Dong Y, Cui Y, Sun S, et al. Telmisartan and Rosuvastatin Synergistically Ameliorate Dementia and Cognitive Impairment in Older Hypertensive Patients With Apolipoprotein E Genotype. Front Aging Neurosci. 2020;12:154.

8. Hwang D, Kim S, Choi H, Oh I-H, Kim BS, Choi HR, et al. Calcium-Channel Blockers and Dementia Risk in Older Adults–National Health Insurance Service–Senior Cohort (2002–2013)–. Circulation Journal. 2016;80(11):2336-42.

9. Li NC, Lee A, Whitmer RA, Kivipelto M, Lawler E, Kazis LE, et al. Use of angiotensin receptor blockers and risk of dementia in a predominantly male population: prospective cohort analysis. Bmj. 2010;340:b5465.

10. Lithell H, Hansson L, Skoog I, Elmfeldt D, Hofman A, Olofsson B, et al. The Study on Cognition and Prognosis in the Elderly (SCOPE): principal results of a randomized double-blind intervention trial. Journal of hypertension. 2003;21(5):875-86.

11. Marcum ZA, Cohen JB, Zhang C, Derington CG, Greene TH, Ghazi L, et al. Association of antihypertensives that stimulate vs inhibit types 2 and 4 angiotensin II receptors with cognitive impairment. JAMA network open. 2022;5(1):e2145319-e.

12. Marcum ZA, Gabriel N, Bress AP, Hernandez I. Association of New Use of Antihypertensives That Stimulate vs Inhibit Type 2 and 4 Angiotensin II Receptors With Dementia Among Medicare Beneficiaries. JAMA Netw Open. 2023;6(1):e2249370.

13. Schroevers JL, Eggink E, Hoevenaar-Blom MP, Van Dalen JW, Van Middelaar T, Van Gool WA, et al. Antihypertensive medication classes and the risk of dementia over a decade of follow-up. J Hypertens. 2023;41(2):262-70.

14. Schroevers JL, Hoevenaar-Blom MP, Busschers WB, Hollander M, Van Gool WA, Richard E, et al. Antihypertensive medication classes and risk of incident dementia in primary care patients: a longitudinal cohort study in the Netherlands. Lancet Reg Health Eur. 2024;42:100927.

15. Tzourio C, Anderson C, Chapman N, Woodward M, Neal B, MacMahon S, et al. PROGRESS Collaborative Group. Effects of blood pressure lowering with perindopril and indapamide therapy on dementia and cognitive decline in patients with cerebrovascular disease. Arch Intern Med. 2003;163(9):1069-75.

16. van Dalen JW, Marcum ZA, Gray SL, Barthold D, Moll van Charante EP, van Gool WA, et al. Association of angiotensin II–stimulating antihypertensive use and dementia risk: post hoc analysis of the PreDIVA trial. Neurology. 2021;96(1):e67-e80.

17. Whiteley WN, Gupta AK, Godec T, Rostamian S, Whitehouse A, Mackay J, et al. Long-Term Incidence of Stroke and Dementia in ASCOT. Stroke. 2021;52(10):3088-96.

18. Yasar S, Corrada M, Brookmeyer R, Kawas C. Calcium channel blockers and risk of AD: the Baltimore Longitudinal Study of Aging. Neurobiol Aging. 2005;26(2):157-63.
